# Harnessing Case Isolation and Ring Vaccination to Control Ebola

**DOI:** 10.1371/journal.pntd.0003794

**Published:** 2015-05-29

**Authors:** Chad Wells, Dan Yamin, Martial L. Ndeffo-Mbah, Natasha Wenzel, Stephen G. Gaffney, Jeffrey P. Townsend, Lauren Ancel Meyers, Mosoka Fallah, Tolbert G. Nyenswah, Frederick L. Altice, Katherine E. Atkins, Alison P. Galvani

**Affiliations:** 1 Center for Infectious Disease Modeling and Analysis, Yale School of Public Health, New Haven, Connecticut, United States of America,; 2 Department of Biostatistics, Yale School of Public Health, New Haven, Connecticut, United States of America,; 3 Program in Computational Biology and Bioinformatics, Yale University, New Haven, Connecticut, United States of America,; 4 Department of Ecology and Evolutionary Biology, Yale University, New Haven, Connecticut, United States of America,; 5 Department of Integrative Biology, University of Texas at Austin, Austin, Texas, United States of America,; 6 Santa Fe Institute, Santa Fe, New Mexico, United States of America,; 7 Ministry of Health and Social Welfare, Monrovia, Liberia,; 8 Section of Infectious Diseases, Yale University School of Medicine, New Haven, Connecticut, United States of America,; 9 Department of Epidemiology of Microbial Diseases, Yale School of Public Health, New Haven, Connecticut, United States of America,; 10 Centre for Mathematical Modelling of Infectious Diseases, Department of Infectious Disease Epidemiology, London School of Hygiene and Tropical Medicine, London, United Kingdom; Tulane School of Public Health and Tropical Medicine, UNITED STATES

## Abstract

As a devastating Ebola outbreak in West Africa continues, non-pharmaceutical control measures including contact tracing, quarantine, and case isolation are being implemented. In addition, public health agencies are scaling up efforts to test and deploy candidate vaccines. Given the experimental nature and limited initial supplies of vaccines, a mass vaccination campaign might not be feasible. However, ring vaccination of likely case contacts could provide an effective alternative in distributing the vaccine. To evaluate ring vaccination as a strategy for eliminating Ebola, we developed a pair approximation model of Ebola transmission, parameterized by confirmed incidence data from June 2014 to January 2015 in Liberia and Sierra Leone. Our results suggest that if a combined intervention of case isolation and ring vaccination had been initiated in the early fall of 2014, up to an additional 126 cases in Liberia and 560 cases in Sierra Leone could have been averted beyond case isolation alone. The marginal benefit of ring vaccination is predicted to be greatest in settings where there are more contacts per individual, greater clustering among individuals, when contact tracing has low efficacy or vaccination confers post-exposure protection. In such settings, ring vaccination can avert up to an additional 8% of Ebola cases. Accordingly, ring vaccination is predicted to offer a moderately beneficial supplement to ongoing non-pharmaceutical Ebola control efforts.

## Author Summary

Public health efforts for controlling the 2014–2015 Ebola outbreak in West Africa have focused on contact tracing and isolation of symptomatic individuals. In addition, substantial resources have been committed to scaling up the production of experimental vaccines. Ring vaccination—the vaccination of the contacts of an infected individual—was successfully implemented to achieve smallpox eradication. Ring vaccination is particularly feasible and effective in settings where the supply of vaccines is limited and disease incidence is low. Using a disease transmission model, we evaluated the benefit of adding ring vaccination to case isolation in Liberia and Sierra Leone. We found that ring vaccination could have averted up to 126 cases in Liberia and 560 cases in Sierra Leone, thereby saving lives and intervention resources.

## Introduction

The Ebola outbreak in West Africa has resulted in unprecedented morbidity and mortality. As of March 4 2015, the World Health Organization (WHO) had reported 23,914 cases and 9,792 fatalities in countries with widespread transmission [1], with Liberia and Sierra Leone having been most profoundly impacted.

Ebola transmission occurs via direct human-to-human contact with body fluids from symptomatic patients. An elevated viral load in late-stage symptomatic or deceased victims can also put family members and funeral attendees at risk of post-mortem disease transmission [2, 3]. The health ministries in Liberia and Sierra Leone have been implementing intensive contact-tracing procedures, where patients or their relatives are interviewed to identify people with whom they came into close contact after developing symptoms. Contacts who are healthy but might have been exposed are monitored for 21 days, the maximum duration of the Ebola incubation period [4]. Contacts who present with Ebola symptoms, such as fever, are transported to isolation clinics [5]. Given that Ebola is transmitted directly between close contacts, the social clustering of individuals can be fundamental to the success of intervention strategies [6–8].

Several Ebola vaccine candidates have been developed in the past decade [9, 10], some of which have already been found to be safe and immunogenic in Phase 1 clinical trials [11]. One of these, a recombinant vesicular stomatitis viruses (rVSV) vaccine, conferred protection to non-human primates when administered immediately following exposure to an otherwise lethal dose of Ebola virus [12]. An alternate vaccine formula based on the chimpanzee adenovirus type 3 (ChAd3), developed in partnership between the National Institute of Allergy and Infectious Disease and GlaxoSmithKline, together with the rVSV vaccine are currently entering Phase 2/3 clinical trials in West Africa [13]. In addition, the WHO has deemed it ethical to use experimental vaccines in the current Ebola emergency situation [14].

Even with the scale up in production [15], the supply would be insufficient for mass vaccination of affected countries, given a combined population of over twenty million people. Consequently, the judicious prioritization of vaccine recipients is essential to maximize vaccine impact. Aside from vaccinating healthcare workers who are at high occupational risk of contracting Ebola [16, 17], a vaccination strategy to reduce community-wide transmission has yet to be evaluated. Evaluating Ebola vaccination strategies is pertinent not only to the current epidemic, but also in mitigating future outbreaks.

The targeting of the exposed contacts of infected individuals, a strategy known as ring vaccination [18, 19], is efficient for controlling rare pathogens [20]. For example, ring vaccination proved to be an effective strategy for smallpox eradication [18, 19]. Furthermore, ring vaccination could be seamlessly incorporated into the contact tracing efforts underway in the affected countries.

To evaluate the effectiveness of Ebola ring vaccination in West Africa, we developed a mathematical model that approximates disease progression in a realistic contact network. We predicted that the marginal benefit was greatest in settings where there are more contacts per individual, greater clustering, or insufficient resources for effective contact tracing. However, we found that ring vaccination provides moderate marginal benefit beyond current non-pharmaceutical interventions.

## Methods

### Ebola transmission model

**Fig 1:**
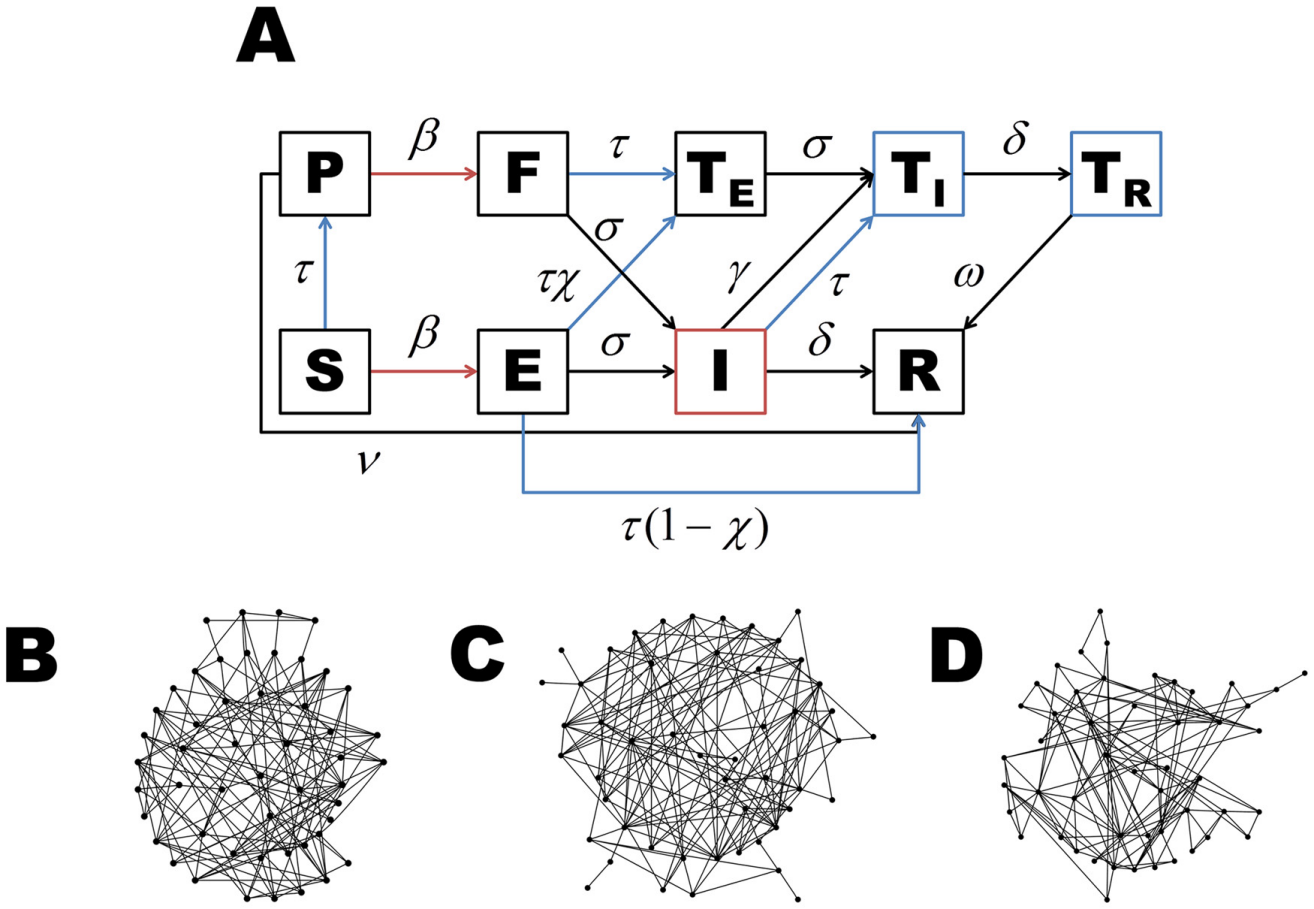
A) Our dynamic model is driven by the spatial correlation of individuals in the population. New latent infections depend on the connections between susceptible and infectious individuals (red). Case isolation and ring vaccination depend on the connections between individuals in the general population (i.e. *S*, *E*, and *I*) and those in isolation (*T*_*I*_ and *T*_*R*_) (blue). B)-D) Examples of networks with an average of 5.5 contacts per individual (approximating the 5.74 estimate from Liberia [22]) and clustering coefficients of B) 0.10, C) 0.21, and D) 0.40.

To determine the effectiveness of ring vaccination and case isolation of Ebola in West Africa, we modeled the transmission between close contacts by using the pair-approximation methodology which simulates disease propagation through a network (Fig 1, S1 Text) [6, 21]. Specifically, we tracked both the number of susceptible individuals ([*S*]), latently infected individuals ([*E*]), infectious individuals ([*I*]), removed individuals ([*R*]), as well as the number of contacts between epidemiological states. For example, [*SI*] denotes the number of contacts between susceptible and infectious individuals. We denoted the average number of contacts per individual in the network by *k*. To account for empirical mixing patterns between individuals during the Ebola outbreak in West Africa [22], we considered clustering of individuals. Clustering can be defined as the extent to which individuals who are in contact with each other share other contacts in the network and is quantified by the clustering coefficient (*ϕ*).

A susceptible individual becomes latently infected (*E*) at rate *β*[*SI*] per day, where *β* is the transmission rate. An individual remains in the latent period for an average duration of 1/*σ* days until becoming symptomatic and infectious (*I*). An infectious individual will transition to the removed state (*R*) (*i.e.* recovered or deceased) at rate *δ*, where 1/*δ* days is the average duration of the infectious period. In addition, we incorporated case isolation by removing a percentage of infectious individuals (*ψ*) from the community at rate *γ* per day (Table 1).

**Table 1:** Epidemiological parameters used in dynamic model.

Parameter	Description (unit)	Value (SA Value)	Reference
*N*	Population size of Liberia	4,092,310	[51]
*N*	Population size of Sierra Leone	6,348,350	[52]
1/*δ*	Average duration of infectious period (days)	12	[53–55]
1/*σ*	Average duration of latent period (days)	9	[53, 56]
*ϕ*	Clustering coefficient	0.21 (0.10 and 0.40)	[22–24]
*k*	Average number of contacts	5.74 (10)	[22]
*E*_0_	Initial number of exposed individuals	2 (Liberia)	
		14 (Sierra Leone)	[57]
1/*ω*	The duration of follow up of contacts (days)	21	[58]
1/*ν*	The average serial interval (days)	15	[53, 55]
1/*υ*	The average time to vaccine acquired immunity (days)	14	[59–62]
1/*γ*	The average number of days until infected individuals enter isolation (days)	5	[53, 63]
Ψ	The fraction of infected individuals that enter isolation	80%	[53, 63]
	$\psi =\frac{\Psi \delta }{(1-\Psi )\gamma +\Psi \delta }$	0.625	[64]

For our base case analysis, we used *k* = 5.74 as the mean number of contacts and *ϕ* = 0.21 for the clustering coefficient, derived from contact tracing data collected by the Liberian Ministry of Health and Social Welfare [22]. We also considered clustering coefficients of 0.10 and 0.40 (Table 1), consistent with previous studies on human contact networks [23–26]. In addition, we accounted for possible under-reporting of contacts by considering higher values of *k* (Table 1).

### Contact tracing and case isolation

During contact tracing, an infected contact in the latent period moves to the observed state (*T*_*E*_) while a contact in the symptomatic period enters the isolated state (*T*_*I*_) at a daily contact tracing rate of *τ* per isolated case. Once an isolated individual has recovered and is no longer infectious, they transition to the *T*_*R*_ state (S1 Text), while the individuals contacts are followed for an additional 1/*ω* days (Table 1). We defined the contact tracing efficacy by the probability of identifying an infected individual before transmission occurs (*τ*/(*τ*+*ν*)), where 1/*ν* is the average serial interval (Table 1). We assumed that the base case contact tracing efficacy was 40%, consistent with empirical estimates [27, 28]. We deemed the current Ebola epidemic to be eliminated at the point where incidence became lower than 0.025 cases per day, which corresponds to no new cases over a 42 day period [29].

$$\begin{array}{ccc} \frac{\text{d}[S]}{\text{d}t} & = & -\beta [SI]\\ \frac{\text{d}[E]}{\text{d}t} & = & \beta \left[SI\right]-\tau \left(\left[ET_{I}\right]+\left[ET_{R}\right]\right)-\sigma \left[E\right]\\ \frac{\text{d}[I]}{\text{d}t} & = & \sigma \left[E\right]-\left(1-\psi \right)\delta \left[I\right]-\psi \gamma \left[I\right]-\tau (\left[IT_{I}\right]+\left[IT_{R}\right])\\ \frac{\text{d}[R]}{\text{d}t} & = & \left(1-\psi \right)\delta \left[I\right]+\omega \left[T_{R}\right]\\ \frac{\text{d}[T_{E}]}{\text{d}t} & = & \tau \left(\left[ET_{I}\right]+\left[ET_{R}\right]\right)-\sigma \left[T_{E}\right]\\ \frac{\text{d}[T_{I}]}{\text{d}t} & = & \tau \left(\left[IT_{I}\right]+\left[IT_{R}\right]\right)-\delta \left[T_{I}\right]+\sigma \left[T_{E}\right]+\psi \gamma \left[I\right]\\ \frac{\text{d}[T_{R}]}{\text{d}t} & = & \delta \left[T_{I}\right]-\omega \left[T_{R}\right]\\ \frac{\text{d}[SI]}{\text{d}t} & = & -\beta (\left[SI\right]+\left(1-\phi \right)\frac{k-1}{k}\frac{{\left[SI\right]}^{2}}{\left[S\right]}+\phi \frac{k-1}{k}\frac{N}{k}\frac{{\left[SI\right]}^{2}\left[II\right]}{{\left[I\right]}^{2}\left[S\right]})\\ & - & \;\tau (\left(1-\phi \right)\frac{k-1}{k}\frac{\left[T_{I}I\right]\left[SI\right]}{\left[I\right]}+\phi \frac{k-1}{k}\frac{N}{k}\frac{\left[T_{I}I\right]\left[SI\right]\left[ST_{I}\right]}{\left[S\right]\left[I\right]\left[T_{I}\right]})\\ & - & \;\tau (\left(1-\phi \right)\frac{k-1}{k}\frac{\left[T_{R}I\right]\left[SI\right]}{\left[I\right]}+\phi \frac{k-1}{k}\frac{N}{k}\frac{\left[T_{R}I\right]\left[SI\right]\left[ST_{R}\right]}{\left[S\right]\left[I\right]\left[T_{R}\right]})\\ & + & \;\sigma [SE]-(1-\psi )\delta [SI]-\psi \gamma [SI] \end{array}$$

### Ring vaccination

We assumed that a proportion of the contacts of isolated cases identified through contact tracing are vaccinated. Experimental studies indicate that several vaccine candidates facilitate recovery of latent infection in non-human primates if administered within two days post-infection [12, 30]. We used a vaccine efficacy (*ɛ*) of 100% as our base case scenario and consider a range of vaccine efficacies from 5% to 95% (S1 Text). We investigated two scenarios: 1) vaccination must be administered pre-exposure in order to confer protection such that only susceptible individuals (*S*) who are uninfected can be protectively vaccinated, and 2) vaccination is efficacious with both pre- and post-exposure administration such that vaccine protection can be conferred to individuals in both the susceptible (*S*) and latently infected (*E*) [30]. The use of a post-exposure vaccine in the equations below is represented by *χ* = 1, otherwise *χ* = 0. Susceptible and latently infected individuals are vaccinated at the same daily contact tracing rate *τ* per isolated individual (S1 Text). When a susceptible individual is vaccinated they remain unprotected (*P*) until vaccine-mediated immunity is acquired 1/*υ* days later.

$$\begin{array}{ccc} \frac{\text{d}[S]}{\text{d}t} & = & -\beta \left[SI\right]-\tau (\left[ST_{I}\right]+\left[ST_{R}\right])\\ \frac{\text{d}[E]}{\text{d}t} & = & \beta \left[SI\right]-\tau \left(\left[ET_{I}\right]+\left[ET_{R}\right]\right)-\sigma \left[E\right]\\ \frac{\text{d}[I]}{\text{d}t} & = & \sigma \left[E\right]+\sigma \left[F\right]-\left(1-\psi \right)\delta \left[I\right]-\tau \left(\left[IT_{I}\right]+\left[IT_{R}\right]\right)-\psi \gamma \left[I\right]\\ \frac{\text{d}[R]}{\text{d}t} & = & \left(1-\psi \right)\delta \left[I\right]+\chi \tau \left(\left[ET_{I}\right]+\left[ET_{R}\right]\right)+\omega \left[T_{R}\right]+\upsilon \left[P\right]\\ \frac{\text{d}[P]}{\text{d}t} & = & \tau \left(\left[ST_{I}\right]+\left[ST_{R}\right]\right)-\beta \left[PI\right]-\upsilon \left[P\right]\\ \frac{\text{d}[F]}{\text{d}t} & = & \beta \left[PI\right]-\tau \left(\left[FT_{I}\right]+\left[FT_{R}\right]\right)-\sigma \left[F\right]\\ \frac{\text{d}[T_{E}]}{\text{d}t} & = & \left(1-\chi \right)\tau \left(\left[ET_{I}\right]+\left[ET_{R}\right]\right)+\tau \left(\left[FT_{I}\right]+\left[FT_{R}\right]\right)-\sigma \left[T_{E}\right]\\ \frac{\text{d}[T_{I}]}{\text{d}t} & = & \tau \left(\left[IT_{I}\right]+\left[IT_{R}\right]\right)-\delta \left[T_{I}\right]+\sigma \left[T_{E}\right]+\psi \gamma \left[I\right]\\ \frac{\text{d}[T_{R}]}{\text{d}t} & = & \delta \left[T_{I}\right]-\omega \left[T_{R}\right] \end{array}$$

### Epidemiological data and parameter estimates

To account for the recent decline in Ebola incidence [31, 32], we used a piecewise approach to calibrate our model, fitting both the early growth phase of the epidemic and the later phase of reduced transmission (S1 Fig, S1 Table, S1 Text). We estimated the rate of transmission per infectious contact (*β*), the date of Ebola emergence into the population (*t*_0_), the initiation of intervention scale up (*t*_*S*_), and reduced transmission mediated by factors other than intervention, such as behavior change (*ξ*). From the date of intervention scale up, we assumed that case isolation was implemented at our base case contact tracing efficacy. Using a least squares fitting algorithm, we estimated these four parameters from weekly confirmed incidence data in Liberia and Sierra Leone, respectively (S1 Table and S1 Text). We fit the model to confirmed incidence data from June 8, 2014 to January 4, 2015 for Liberia [31] and from May 11, 2014 to January 4, 2015 for Sierra Leone [32] (S1 Table and S1 Text). However, on December 20, 2014, the deployment of international aid began in Sierra Leone and considerably reduced transmission [33–35]. Thus, we considered a second reduction in transmission from this time point (S1 Text). To validate the calibration of Liberia and Sierra Leone, we forecasted the incidence of Ebola to March 8, 2015 and calculated the correlation fit value to the observed incidence (S1 Fig, S1 Table).

## Results

### Base case analysis

**Fig 2:**
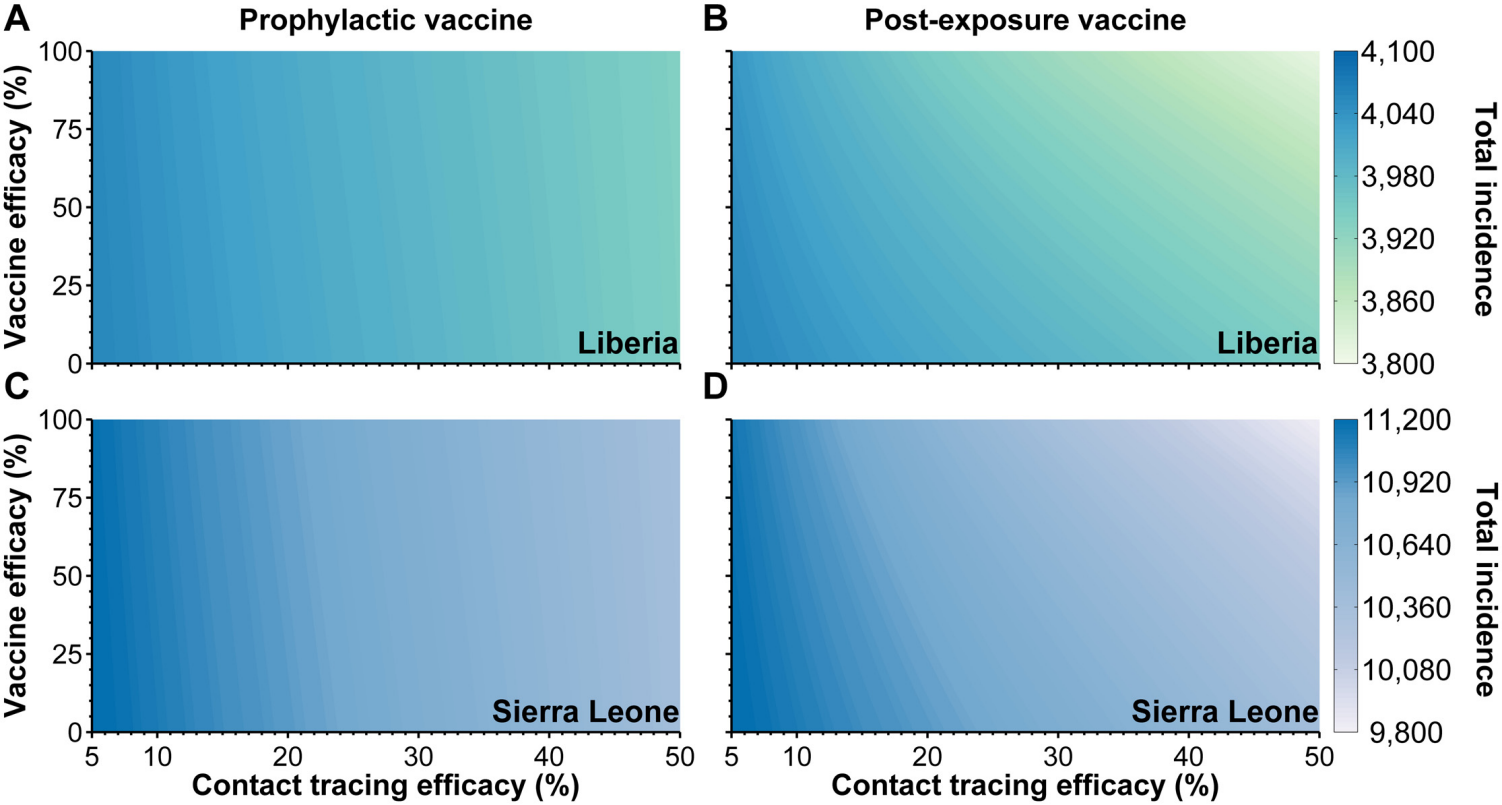
The estimated total number of confirmed cases for various contact tracing and vaccine efficacies in (A)-B)) Liberia and (C)-D)) Sierra Leone using (A), C)) a prophylactic vaccine and (B), D)) a vaccine that confers post-exposure protection. The model was fit using *k* = 5.74 with a clustering coefficient of *ϕ* = 0.21, as estimated for Liberia [22]. A vaccine efficacy of zero would correspond to the implementation of case isolation only.

We estimated that intervention efforts were initially scaled up on September 5, 2014 in Liberia and October 8, 2014 in Sierra Leone. Our model predicts that under status quo intervention the total number of confirmed Ebola cases in Liberia will be 3,899 cases and 10,425 cases in Sierra Leone (Fig 2). If ring vaccination using a prophylactic vaccine had been combined with the initial scaling up of non-pharmaceutical interventions, four additional cases could have been averted in Liberia and 36 cases could have been averted in Sierra Leone, corresponding to relatively low marginal benefits of 0.21% and 0.48% additional cases averted, respectively (S2 Fig). By contrast, for a vaccine that can confer post-exposure protection, we estimate that 107 cases could have been averted in Liberia and 477 cases could be averted in Sierra Leone (Fig 2), corresponding to marginal benefits of 4.7% and 6.5%, respectively (S3 Fig).

We found that a prophylactic vaccine had minimal impact on the number of symptomatic individuals identified by contact tracing (S4 Fig). However, if the vaccine confers post-exposure protection, then ring vaccination is predicted to reduce the number of symptomatic individuals identified by contact tracing by 40 in Liberia and 137 in Sierra Leone compared to case isolation alone, thereby reducing requirements of isolation units in hospitals (S4 Fig).

### Sensitivity analysis

We conducted sensitivity analysis with respect to contact tracing efficacy, average number of contacts per individuals (*k*), and the clustering coefficient (*ϕ*) (S2 Text). Varying contact tracing efficacy from 5% to 50%, the predicted epidemic size without ring vaccination ranged from 3,874 to 3,988 confirmed cases in Liberia and 10,246 to 11,048 confirmed cases in Sierra Leone (Fig 2). Implementing ring vaccination with a prophylactic vaccine is expected to achieve the greatest marginal benefit when contact tracing efficacies are between 20% and 28% in both Liberia and Sierra Leone (S2 Fig, S2 Text). Specifically, the greatest marginal benefit of ring vaccination can be up to 0.29% in Liberia and up to 0.64% in Sierra Leone, depending on the vaccine efficacy (S2 Fig). However, a vaccine with post-exposure protection is estimated to provide up to 6% marginal benefit in Liberia and 8% in Sierra Leone (S2 Text, S3 Fig). These results suggest that ring vaccination provides moderate benefit to case isolation under a range of contact tracing efficacies and vaccine efficacies.

Our sensitivity analysis of the social mixing patterns (*k* and *ϕ*) show that the marginal benefit of adding ring vaccination rises with more contacts per individual or increasing clustering (S2 and S5 Figs, S2 Text).

## Discussion

We demonstrated that a combination of contact tracing, case isolation and ring vaccination could effectively reduce Ebola transmission. In Liberia, as well as in past Ebola outbreaks, implementing case isolation along with change in human behavior dramatically reduced transmission [36–39], which commonly occurs when people perceive infection risk associated with specific behaviors. Our results suggest that in this current context of reduced transmission, ring vaccination offers only moderate additional benefit, which is consistent with findings from a previous cholera model [40]. Although the incremental benefit of ring vaccination could be relatively moderate, the marginal benefit of ring vaccination is expected to increase with the average number of contacts per individual, the clustering coefficient or lower contact tracing efficacies. Therefore, ring vaccination is particularly useful in regions where contact tracing is logistically challenging. In addition, a vaccine that can confer post-exposure efficacy is much more effective for use in ring vaccination strategies than a vaccine that can only confer protection prophylactically. An additional benefit of ring vaccination that is more pronounced for a vaccine that confers post-exposure protection than for an exclusively prophylactic vaccine is the reduction in the number of symptomatic individuals who need hospitalization.

The 2014 Ebola outbreak in West Africa has affected both rural and urban communities [41], which differ in accessibility for contact tracing and hospital facilities for case isolation. There is also regional variation in the number and clustering of contacts [42]. Our analysis demonstrates that the benefit of adding ring vaccination to case isolation is influenced by the extent of clustering in a population. Specifically, we found ring vaccination provided the greatest marginal benefit to case isolation in highly clustered populations, such as crowded homes or schools [23–26, 43]. As social networks can be highly complex systems consisting of densely connected communities [44–46], future studies should evaluate not only transmission patterns within communities but also transmission patterns between rural and urban areas in order to tailor ring vaccination to specific locations.

Clinical trials are underway to evaluate Ebola vaccine candidates, but there is currently considerable uncertainty regarding the efficacy post-exposure and prophylactically. Our analysis of vaccine efficacy, ranging between 5–100%, demonstrates a diminishing marginal contribution with declining vaccine efficacy. For example, at a hypothetical efficacy of 50% ring vaccination could have averted up to 214 cases, whereas a vaccine with an efficacy of 75% up to 360 cases of Ebola could have been averted.

Our modeling approach is based on a deterministic approximation to a stochastic network model. Consequently, our model did not capture the stochastic effects that become pronounced in the eradication phase of an outbreak [6] and for ring vaccination strategies that include second-order ring vaccination, where contacts of exposed individuals are vaccinated in addition to the exposed contacts of the isolated case. We would expect an increase in the marginal benefit of ring vaccination if both first-order and second-order vaccination were implemented because vaccination would be administered ahead of the wave of transmission. Thus, our results are conservative with regard to second-order ring vaccination.

Ebola has similar family and household transmission as smallpox. During the final eradication phase of smallpox, public health authorities relied on case isolation and ring vaccination [18, 19, 47, 48]. Because smallpox is more infectious than Ebola [2, 49, 50], we conservatively expect that case isolation and ring vaccination would likewise be a practical approach to eliminate Ebola and contain future outbreaks, especially with limited vaccine supplies.

The combined implementation of ring vaccination and case isolation can be an effective approach in curtailing an Ebola outbreak. Our model predicts that ring vaccination offers moderate benefit to case isolation, with the greatest benefit occurring where there are more contacts per individual or greater clustering among individuals, and when contact tracing has low efficacy or vaccination can confer post-exposure protection.

## Supporting Information

S1 Text Model description and equations. (PDF)

S2 Text Extension of sensitivity analysis regarding mixing patterns and vaccine characteristics. (PDF)

S1 Fig The estimated weekly confirmed incidence for (left column) Liberia and (right column) Sierra Leone up to March 8, 2015 (black line) for A)-B) *k* = 5.74 and *ϕ* = 0.21, C)-D) *k* = 5.74 and *ϕ* = 0.10, E)-F) *k* = 5.74 and *ϕ* = 0.40, and G)-H) *k* = 10 and *ϕ* = 0.21. The model was fit to confirmed incidence data (black dots) from June 8, 2014 to January 4, 2015 for Liberia and May 11, 2014 to January 4, 2015 for Sierra Leone. We forecasted confirmed incidence until March 8, 2015 (red points). For each fit, we calculated the correlation fit value (*R* ^2^) for the fitted portion (black) and the forecasted portion (red) of the data.
(TIFF)

S2 Fig The estimated marginal benefit of adding ring vaccination to case isolation for various efficacies of contact tracing and the vaccine in A)-B) Liberia and C)-D)Sierra Leone. The model was fit using A), C) *k* = 5.74 and B), D) *k* = 10, with a clustering coefficient of *ϕ* = 0.21. A vaccine efficacy of zero would correspond to the implementation of case isolation only. The marginal benefit was calculated from the initiation of intervention scale up to the end of the epidemic.
(TIFF)

S3 Fig The estimated marginal benefit of adding ring vaccination to case isolation for various efficacies of contact tracing and the vaccine in A) Liberia and B)Sierra Leone for a vaccine that confers post-exposure protection. The model was fit using *k* = 5.74 with a clustering coefficient of *ϕ* = 0.21. A vaccine efficacy of zero would correspond to the implementation of case isolation only. The marginal benefit was calculated from the initiation of intervention scale up to the end of the epidemic.
(TIFF)

S4 Fig The estimated number of symptomatic cases identified through contact tracing as a result of adding ring vaccination for various efficacies of contact tracing and the vaccine in A)-B) Liberia and C)-D) Sierra Leone using (A), C)) a prophylactic vaccine and (B), D)) a vaccine that confers post-exposure protection. A vaccine efficacy of zero would correspond to the implementation of case isolation only. The model was fit using *k* = 5.74 and with a clustering coefficient of *ϕ* = 0.21.
(TIFF)

S5 Fig The estimated marginal benefit of adding ring vaccination to case isolation for various efficacies of contact tracing and the vaccine in A)-C) Liberia and D)-F) Sierra Leone. The model was fit using *k* = 5.74 with a clustering coefficient of A), D) *ϕ* = 0.10, B), E) *ϕ* = 0.21, and C), F) *ϕ* = 0.40. A vaccine efficacy of zero would correspond to the implementation of case isolation only. The marginal benefit was calculated from the initiation of intervention scale up to the end of the epidemic.
(TIFF)

S6 Fig The estimated marginal benefit of increasing the efficacy of contact tracing and of the vaccine in A)-B) Liberia and C)-D) Sierra Leone. The model was fit using A), C) *k* = 5.74 and B), D) *k* = 10, with a clustering coefficient of *ϕ* = 0.21. A vaccine efficacy of zero would correspond to the implementation of case isolation only. The marginal benefit was calculated from the initiation of intervention scale up to the end of the epidemic.
(TIFF)

S7 Fig The estimated marginal benefit of increasing the efficacy of contact tracing and of the vaccine in A)-C) Liberia and D)-F) Sierra Leone. The model was fit using *k* = 5.74 with a clustering coefficient of A), D) *ϕ* = 0.10, B), E) *ϕ* = 0.21, and C), F) *ϕ* = 0.40. A vaccine efficacy of zero would correspond to the implementation of case isolation only. The marginal benefit was calculated from the initiation of intervention scale up to the end of the epidemic.
(TIFF)

S8 Fig The estimated total number of confirmed cases for various contact tracing and vaccine efficacies in A)-B) Liberia and C)-D) Sierra Leone. The model was fit using A), C) *k* = 5.74 and B), D) *k* = 10, with a clustering coefficient of *ϕ* = 0.21. A vaccine efficacy of zero would correspond to the implementation of case isolation only.
(TIFF)

S9 Fig The estimated number of symptomatic cases identified through contact tracing as a result of adding ring vaccination for various efficacies of contact tracing and the vaccine in A)-B) Liberia and C)-D) Sierra Leone. The model was fit using A), C) *k* = 5.74 and B), D) *k* = 10, with a clustering coefficient of *ϕ* = 0.21. A vaccine efficacy of zero would correspond to the implementation of case isolation only.
(TIFF)

S10 Fig The estimated total number of confirmed cases for various contact tracing and vaccine efficacies in A)-C) Liberia and D)-F) Sierra Leone. The model was fit using *k* = 5.74 with a clustering coefficient of A), D) *ϕ* = 0.10, B), E) *ϕ* = 0.21, and C), F) *ϕ* = 0.40. A vaccine efficacy of zero would correspond to the implementation of case isolation only.
(TIFF)

S11 Fig The estimated number of symptomatic cases identified through contact tracing as a result of adding ring vaccination for various efficacies of contact tracing and the vaccine in A)-C) Liberia and D)-F) Sierra Leone using (A), C)). The model was fit using *k* = 5.74 with a clustering coefficient of A), D) *ϕ* = 0.10, B), E) *ϕ* = 0.21, and C), F) *ϕ* = 0.40. A vaccine efficacy of zero would correspond to the implementation of case isolation only.
(TIFF)

S1 TableEstimated parameters for the dynamic model.Using confirmed incidence data for Liberia and Sierra Leone, we estimated the rate of transmission per infectious contact (*β*), the date of Ebola emergence into the population (*t*_0_), the initiation of intervention scale up (*t*_*S*_), and reduced transmission mediated by factors other than intervention (*ξ*) for a given average number of contacts (*k*) and clustering coefficient (*ϕ*). For each scenario we provide the basic reproductive number (*R*_0_), the mean square error (MSE), and the correlation fit value for both the fitted portion (*R* ^2^) and the forecasted portion ($R_{F}^{2}$) of the epidemic trajectory.(TIFF)
