# Correction: Harnessing Case Isolation and Ring Vaccination to Control Ebola

**DOI:** 10.1371/journal.pntd.0003888

**Published:** 2015-06-24

**Authors:** Chad Wells, Dan Yamin, Martial L. Ndeffo-Mbah, Natasha Wenzel, Stephen G. Gaffney, Jeffrey P. Townsend, Lauren Ancel Meyers, Mosoka Fallah, Tolbert G. Nyenswah, Frederick L. Altice, Katherine E. Atkins, Alison P. Galvani

There are two errors in Fig 1. In Fig 1, the label for the edge connecting the latent state (E) to the removed state (R) should be τχ. The label for the edge connecting the latent state (E) to the observed state (T_E_) should be τ(1-χ). Please see the corrected Fig 1 here.

**Fig 1:**
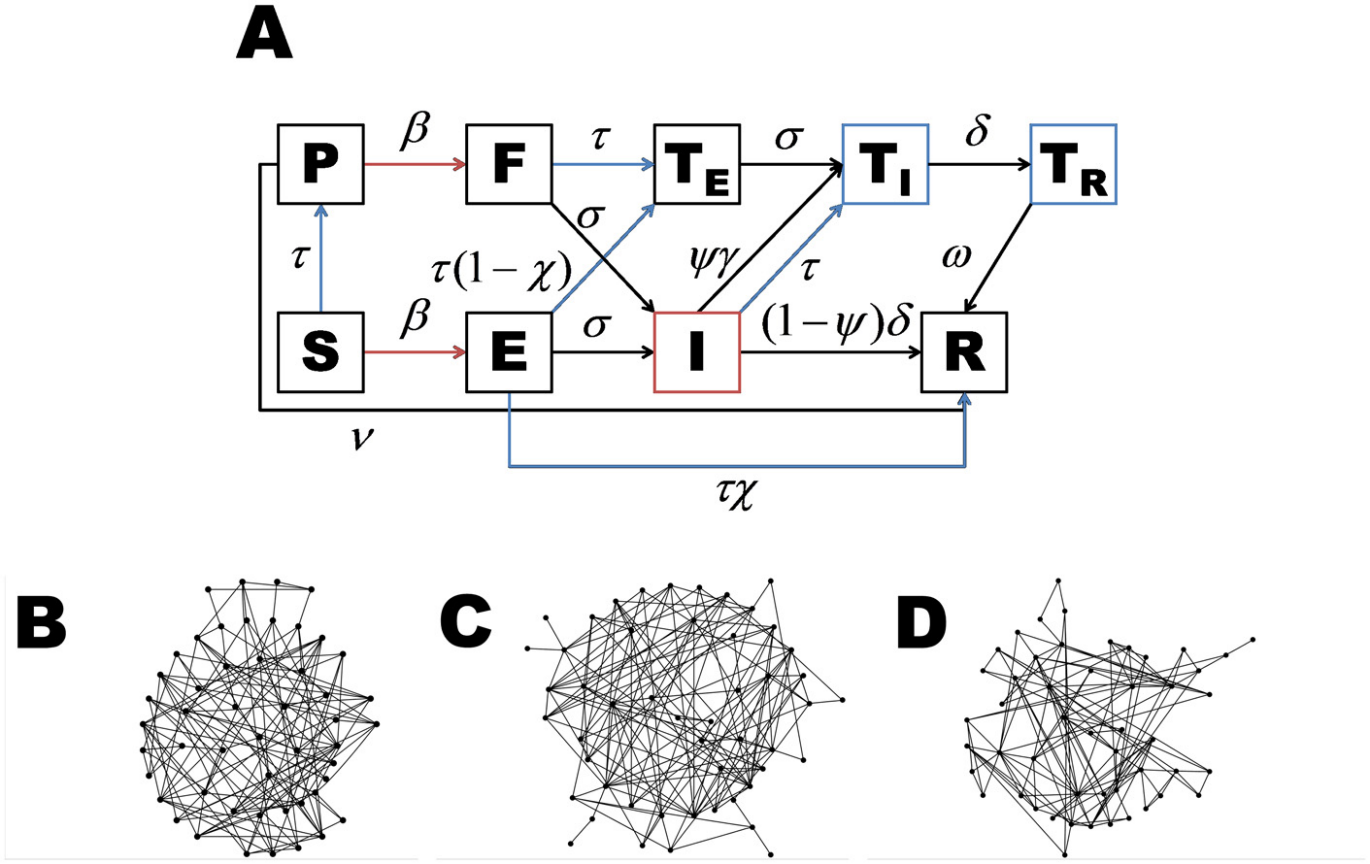
**A) Our dynamic model is driven by the spatial correlation of individuals in the population.** New latent infections depend on the connections between susceptible and infectious individuals (red). Case isolation and ring vaccination depend on the connections between individuals in the general population (i.e. *S*, *E*, and *I*) and those in isolation (*T*_*I*_ and *T*_*R*_) (blue). B)-D) Examples of networks with an average of 5.5 contacts per individual (approximating the 5.74 estimate from Liberia [22]) and clustering coefficients of B) 0.10, C) 0.21, and D) 0.40.
