# Supplementary material for: Harnessing Case Isolation and Ring Vaccination to Control Ebola
Source: PLoS Negl Trop Dis. 2015 May 29;9(5):e0003794. doi: 10.1371/journal.pntd.0003794 (PMC4449200; doi:10.1371/journal.pntd.0003794)
Supplement: S1 Text — (PDF) [file pntd.0003794.s001.pdf]

Text S1: Model description and equations

Chad Wells, Dan Yamin, Martial L. Ndeffo-Mbah, Natasha Wenzel, Stephen G. Gaffney, Jeffrey P. Townsend, Lauren Ancel Meyers, Mosoka Fallah, Tolbert G. Nyenswah, Frederick L. Altice, Katherine E. Atkins, and Alison P. Galvani

Model

We modeled the transmission of Ebola based on a Susceptible-Exposed-Infected-Removed ($SEIR$) natural history of infection [1–4] using pair approximations. Pair-approximation models are formulated in terms of a hierarchy of equations where first-order moments, such as $[S]$, depend on second-order moments, such as $[SI]$, which then depend on third-order moments, such as $[ISI]$. The average degree of the network is defined as the average number of contacts per individual in the population and is denoted by k. The clustering coefficient is a measure of the number of shared contacts between neighbors in the population and is denoted by ϕ. Approximating the higher moments collapses the hierarchy of equations such that the density of triplets, or third order moments, depends only on the density of pairs. Consistent with previous models on clustered populations [5], to close the system we used the standard triangle pair approximation:

$$
\begin{aligned}
[ijm] &= (1-\phi)\frac{k-1}{k}\frac{[ij][jm]}{[j]} + \phi\frac{k-1}{k}\frac{N}{k}\frac{[ij][jm][im]}{[i][j][m]} \\
[iji] &= [ij] + (1-\phi)\frac{k-1}{k}\frac{[ij][ji]}{[j]} + \phi\frac{k-1}{k}\frac{N}{k}\frac{[ij][ji][ii]}{[i][j][i]},
\end{aligned}
$$

where N is the population size, k is the average degree, ϕ is the clustering coefficient, and i, j, and m can be any of the epidemiological states.

Infection may occur when a susceptible, S, has contact with an infectious individual I, which is represented by the density of susceptible-infected pairs $[SI]$. A susceptible individual transitions into the latent state, E, at rate $\beta[SI]$, where β is the transmission rate. A latently infected individual becomes infectious after $1/\sigma$ days. An individual remains infectious for an average of $1/\delta$ days, where ψ percent of the infected individuals enter case isolation via self-reporting (as opposed to active contact tracing) at a rate γ per day. Our criteria for Ebola elimination is a threshold of fewer than 0.025 cases per day, which corresponds to approximately one new case over a 42 day duration [6].

Contact tracing and Case isolation

Following contact with an isolated individual who is or was symptomatic (T_I and T_R), latently infected (E) and symptomatically infectious individuals (I) are traced at a rate τ, such that they are either under observation (T_E) or, if they become symptomatic after $1/\sigma$ days, will be isolated (T_I). An individual in isolation enters the removed traced state after $1/\delta$ days when the infectious period ends, resulting either in death or recovery ($T_I \rightarrow T_R$). We keep track of the removed state in order to model the continued tracing of contacts of formerly isolated individuals for a period of 21 days, which corresponds to the maximum duration of the latent phase [7].

Ring vaccination

We assumed that a proportion of contacts identified through contact tracing are vaccinated. To incorporate imperfect vaccine efficacy, we assumed that ε of the population obtain full protective immunity from vaccination (S) while the remaining population (S_N) is not protected. We denote latently infected individuals who were previously vaccinated and latently infected individuals who do not obtain protective

immunity by F. We considered vaccine efficacies ranging from 5% to 100%, corresponding to the initial conditions:

$$\begin{aligned}
[E](0) &= \varepsilon E_0 \\
[F](0) &= (1-\varepsilon)E_0 \\
[S](0) &= \varepsilon(N-[E](0)-[F](0)) \\
[S_N](0) &= (1-\varepsilon)(N-[E](0)-[F](0)) \\
[SS_N](0) &= \varepsilon(1-\varepsilon)k(N-[E](0)-[F](0)) \\
[SE](0) &= \varepsilon k[E](0) \\
[S_NE](0) &= (1-\varepsilon)k[E](0) \\
[SF](0) &= \varepsilon k[F](0) \\
[S_NF](0) &= (1-\varepsilon)k[F](0) \\
[S_NS_N](0) &= (1-\varepsilon)k(N-[E](0)-[F](0))-[SS_N](0)-[S_NE](0)-[S_NF](0) \\
[SS](0) &= \varepsilon k(N-[E](0)-[F](0))-[SS_N](0)-[SE](0)-[SF](0).
\end{aligned}$$

We denote the tracing and vaccination rate by τ and $\chi\tau$ for a susceptible individual and a latently infected individual, respectively. In addition, the use of a prophylactic vaccine is denoted $\chi = 0$ and the use of a vaccine that confers post-exposure protection is denoted $\chi = 1$. When a susceptible individual is vaccinated with the prophylactic vaccine, the individual transitions into an unprotected pre-vaccinated state (P) at a rate of $\tau([ST_I]+[ST_R])$. After $1/\upsilon$ days, protective vaccine-mediated immunity is assumed to have mounted and the vaccinated individual transitions into the removed state. Latently infected individuals who obtain post-exposure immunity after vaccination (E) transition directly to the removed class. If the vaccine does not take, the latently infected individual remain under daily observation (T_E), from which they can still be isolated once they become symptomatic.

Model fitting

For Liberia, we fit the model to the number of weekly confirmed cases reported by the WHO from June 8, 2014 to January 4, 2015 (Figure S1) [8]. Similarly, for Sierra Leone, we fit the number of weekly confirmed cases reported by the WHO between May 11, 2014 and January 4, 2015 (Figure S1) [9]. We calibrated the model by fitting the transmission rate (β), the date of Ebola emergence into the population (t_0), the initiation of intervention scale up (t_S), and the reduction in transmission as a result of external factors (ξ). During the time between disease emergence and the downturn in transmission, we assumed no scale up in the intervention (*i.e.* $\tau = 0$). From the start of intervention scale up onwards, we assumed that case isolation was implemented at our base case contact tracing efficacy (40%) [10,11]. In addition to case isolation, our model calibration indicated that there was a reduction in transmission due to external factors, such as behavior. For Sierra Leone, we found that a second reduction in transmission occurred on December 20, 2014, consistent with the deployment of substantial international aid at that time [12–14], and corresponding to a further ξ reduction in transmission. For each model fit, we used a fixed clustering coefficient (ϕ) of 0.21, 0.10 or 0.40 and an average degree (k) of 5.74 or 10.

The piecewise transmission rate for Liberia is:

$$\beta(t) = \begin{cases} \beta & : t_0 \le t < t_S \\ (1-\xi)\beta & : t_S \le t \end{cases}, \tag{1}$$

and the piecewise transmission rate for Sierra Leone is:

$$\beta(t) = \begin{cases} \beta & : t_0 \leq t < t_S \\ (1-\xi)\beta & : t_S \leq t < \text{Dec. } 20,\ 2014 \\ (1-\xi)^2\beta & : \text{Dec. } 20,\ 2014 \leq t \end{cases}, \tag{2}$$

where $\tau = 0$ for $t < t_S$ and $\tau > 0$ for $t \geq t_S$.

We fit the model to the data for both countries by minimizing the mean square error (Table S1),

$$Error = \frac{1}{N-1}\sum_{i=1}^{N}(Y_i - C_i)^2, \tag{3}$$

where at time point i, C_i is the weekly incidence of the data and Y_i is the weekly incidence predicted by the model.

The minimization was implemented using MATLAB 7.12.0 constrained optimization function lsqnonlin [15]. The ordinary differential equation system was solved using MATLAB ode15s routine. We assumed that the time of disease introduction occurred within 0 to 90 days prior to June 8, 2014 for Liberia and May 11, 2014 for Sierra Leone. The origin of the Sierra Leone outbreak was traced to the funeral of a healer who treated Ebola patients in Guinea [16]. During the funeral proceedings in Koindu, Sierra Leone, 14 individuals were infected by direct contact with the deceased [16]. Therefore, we parameterized the initial number of latently infected individuals to 14 for Sierra Leone. For Liberia, we assumed the outbreak in Liberia was initiated by two infected individuals, diagnosed on June 8, 2014 [8].

Quantitative measures

We measure the marginal benefit of adding ring vaccination to case isolation by the relative reduction in the total number of cases during the period throughout which intervention was scaled up:

$$\text{Marginal Benefit} = \frac{C_I(T, \tau/(\tau+\nu)) - C_R(T, \tau/(\tau+\nu))}{C_I(T, \tau/(\tau+\nu)) - C(t_S)},$$

where $C_I(T, \tau/(\tau+\nu))$ denotes the total number of cases for the entire epidemic when case isolation is implemented with contact tracing efficacy $\tau/(\tau+\nu)$. The total number of cases for the entire epidemic when case isolation and ring vaccination are implemented with contact tracing efficacy $\tau/(\tau+\nu)$ is denoted $C_R(T, \tau/(\tau+\nu))$. The total number of cases on the day of initial intervention scale up is denoted $C(t_S)$.

We measure the marginal benefit of increasing the contact tracing efficacy by the relative reduction in the total number of cases during the period throughout which intervention was scaled up,

$$\text{Marginal Benefit} = \frac{C_R(T, 5\%) - C_R(T, \tau/(\tau+\nu))}{C_R(T, 5\%) - C(t_S)},$$

where $C_R(T, \tau/(\tau+\nu))$ denotes the total number of cases for the entire epidemic when case isolation and ring vaccination are implemented with contact tracing efficacy $\tau/(\tau+\nu)$. The total number of cases at the time of initial intervention scale up is denoted $C(t_S)$.

Equations

The first-order moment equations for a combined intervention.

$$
\begin{aligned}
\frac{\mathrm{d}[S]}{\mathrm{d}t} &= -\beta(t)[SI] - \tau([ST_I] + [ST_R]) \\
\frac{\mathrm{d}[E]}{\mathrm{d}t} &= \beta(t)[SI] - \tau([ET_I] + [ET_R]) - \sigma[E] \\
\frac{\mathrm{d}[I]}{\mathrm{d}t} &= \sigma[E] + \sigma[F] - (1-\psi)\delta[I] - \psi\gamma[I] - \tau([IT_I] + [IT_R]) \\
\frac{\mathrm{d}[R]}{\mathrm{d}t} &= (1-\psi)\delta[I] + \chi\tau([ET_I] + [ET_R]) + \omega[T_R] + \upsilon[P] \\
\frac{\mathrm{d}[T_E]}{\mathrm{d}t} &= (1-\chi)\tau([ET_I] + [ET_R]) + \tau([FT_I] + [FT_R]) - \sigma[T_E] \\
\frac{\mathrm{d}[T_I]}{\mathrm{d}t} &= \psi\gamma[I] + \tau([IT_I] + [IT_R]) - \delta[T_I] + \sigma[T_E] \\
\frac{\mathrm{d}[T_R]}{\mathrm{d}t} &= \delta[T_I] - \omega[T_R] \\
\frac{\mathrm{d}[S_N]}{\mathrm{d}t} &= -\beta(t)[S_N I] \\
\frac{\mathrm{d}[P]}{\mathrm{d}t} &= \tau([ST_I] + [ST_R]) - \beta(t)[PI] - \upsilon[P] \\
\frac{\mathrm{d}[F]}{\mathrm{d}t} &= \beta(t)[S_N I] + \beta(t)[PI] - \tau([FT_I] + [FT_R]) - \sigma[F]
\end{aligned}
$$

The second-order moment equations for a combined intervention containing the third order moments

$$
\begin{aligned}
\frac{\mathrm{d}[SS]}{\mathrm{d}t} &= -2\beta(t)[ISS] - 2\tau([T_I SS] + [T_R SS]) \\
\frac{\mathrm{d}[SE]}{\mathrm{d}t} &= -\beta(t)[ISE] - \tau([T_I SE] + [T_R SE]) \\
&+ \beta(t)[ISS] - \tau([T_I ES] + [T_R ES]) - \sigma[SE] \\
\frac{\mathrm{d}[SI]}{\mathrm{d}t} &= -\beta(t)[ISI] - \tau([T_I SI] + [T_R SI]) \\
&+ \sigma[SE] + \sigma[SF] - (1-\psi)\delta[SI] - \psi\gamma[SI] - \tau([T_I IS] + [T_R IS]) \\
\frac{\mathrm{d}[SR]}{\mathrm{d}t} &= -\beta(t)[ISR] - \tau([T_I SR] + [T_R SR]) \\
&+ (1-\psi)\delta[SI] + \chi\tau([T_I ES] + [T_R ES]) + \upsilon[SP] \\
&+ \omega[T_R S] \\
\frac{\mathrm{d}[ST_E]}{\mathrm{d}t} &= -\beta(t)[IST_E] - \tau([T_I ST_E] + [T_R ST_E]) \\
&+ (1-\chi)\tau([T_I ES] + [T_R ES]) - \sigma[ST_E] + \tau([T_I FS] + [T_R FS]) \\
\frac{\mathrm{d}[ST_I]}{\mathrm{d}t} &= -\beta(t)[IST_I] - \tau([T_I ST_I] + [T_R ST_I]) \\
&+ \tau([T_I IS] + [T_R IS]) - \delta[ST_I] + \psi\gamma[SI] + \sigma[ST_E] \\
\frac{\mathrm{d}[ST_R]}{\mathrm{d}t} &= -\beta(t)[IST_R] - \tau([T_I ST_R] + [T_R ST_R]) \\
&+ \delta[ST_I] - \omega[ST_R] \\
\frac{\mathrm{d}[SS_N]}{\mathrm{d}t} &= -\beta(t)[ISS_N] - \tau([T_I SS_N] + [T_R SS_N]) - \beta(t)[IS_N S] \\
\frac{\mathrm{d}[SP]}{\mathrm{d}t} &= -\beta(t)[ISP] - \tau([T_I SP] + [T_R SP]) + \tau([T_I SS] + [T_R SS]) - \beta(t)[IPS] - \upsilon[SP] \\
\frac{\mathrm{d}[SF]}{\mathrm{d}t} &= -\beta(t)[ISF] - \tau([T_I SF] + [T_R SF]) \\
&= + \beta(t)[IS_N S] + \beta(t)[IPS] - \tau([T_I FS] + [T_R FS]) - \sigma[SF]
\end{aligned}
$$

$$
\begin{aligned}
\frac{\mathrm{d}[EE]}{\mathrm{d}t} &= 2\beta(t)[ISE] - 2\tau([T_IEE] + [T_REE]) - 2\sigma[EE] \\
\frac{\mathrm{d}[EI]}{\mathrm{d}t} &= \beta(t)[ISI] - \tau([T_IEI] + [T_REI]) - \sigma[EI] \\
&+ \sigma[EF] + \sigma[EE] - (1-\psi)\delta[EI] - \psi\gamma[EI] - \tau([T_IIE] + [T_RIE]) \\
\frac{\mathrm{d}[ER]}{\mathrm{d}t} &= \beta(t)[ISR] \\
&- \tau([T_IER] + [T_RER]) - \sigma[ER] \\
&+ (1-\psi)\delta[IE] + \upsilon[EP] \\
&+ \chi\tau([T_IEE] + [T_REE]) + \omega[ET_R] \\
\frac{\mathrm{d}[ET_E]}{\mathrm{d}t} &= \beta(t)[IST_E] - \tau([T_IET_E] + [T_RET_E]) - \sigma[ET_E] \\
&+ (1-\chi)\tau([T_IEE] + [T_REE]) + \tau([T_IFE] + [T_RFE]) - \sigma[ET_E] \\
\frac{\mathrm{d}[ET_I]}{\mathrm{d}t} &= \beta(t)[IST_I]] - \tau([T_IET_I] + [T_RET_I]) - \sigma[ET_I] \\
&+ \psi\gamma[IE] + \tau([T_IIE] + [T_RIE]) - \delta[T_IE] + \sigma[T_EE] \\
\frac{\mathrm{d}[ET_R]}{\mathrm{d}t} &= \beta(t)[IST_R] - \tau([T_IET_R] + [T_RET_R]) - \sigma[ET_R] \\
&+ \delta[T_IE] - \omega[T_RE] \\
\frac{\mathrm{d}[ES_N]}{\mathrm{d}t} &= \beta(t)[ISS_N]] - \tau([T_IES_N] + [T_RES_N]) - \sigma[ES_N] - \beta(t)[IS_NE] \\
\frac{\mathrm{d}[EP]}{\mathrm{d}t} &= \beta(t)[ISP] - \tau([T_IEP] + [T_REP]) - \sigma[EP] \\
&+ \tau([T_ISE] + [T_RSE]) - \beta(t)[IPE] - \upsilon[EP] \\
\frac{\mathrm{d}[EF]}{\mathrm{d}t} &= \beta(t)[ISF] - \tau([T_IEF] + [T_REF]) - \sigma[EF] \\
&+ \beta(t)[IS_NE] + \beta(t)[IPE] - \tau([T_IFE] + [T_RFE]) - \sigma[EF]
\end{aligned}
$$

$$
\begin{aligned}
\frac{\mathrm{d}[II]}{\mathrm{d}t} &= 2\sigma[IE] + 2\sigma[FI] - 2(1-\psi)\delta[II] - 2\psi\gamma[II] - 2\tau([T_I II] + [T_R II]) \\
\frac{\mathrm{d}[IR]}{\mathrm{d}t} &= \sigma[ER] + \sigma[FR] - (1-\psi)\delta[IR] - \psi\gamma[IR] - \tau([T_I IR] + [T_R IR]) \\
&+ (1-\psi)\delta[II] + \upsilon[IP] + \chi\tau([T_I EI] + [T_R EI]) + \omega[IT_R] \\
\frac{\mathrm{d}[IT_E]}{\mathrm{d}t} &= \sigma[ET_E] + \sigma[FT_E] - (1-\psi)\delta[IT_E] - \psi\gamma[IT_E] - \tau([T_I IT_E] + [T_R IT_E]) \\
&+ (1-\chi)\tau([T_I EI] + [T_R EI]) + \tau([T_I FI] + [T_R FI]) - \sigma[IT_E] \\
\frac{\mathrm{d}[IT_I]}{\mathrm{d}t} &= \sigma[ET_I] + \sigma[FT_I] - (1-\psi)\delta[IT_I] - \psi\gamma[IT_I] - \tau([T_I IT_I] + [T_R IT_I]) \\
&+ \psi\gamma[II] + \tau([T_I II] + [T_R II]) - \delta[T_I I] + \sigma[T_E I] \\
\frac{\mathrm{d}[IT_R]}{\mathrm{d}t} &= \sigma[ET_R] + \sigma[FT_R] - (1-\psi)\delta[IT_R] - \psi\gamma[IT_R] - \tau([T_I IT_R] + [T_R IT_R]) \\
&+ \delta[T_I I] - \omega[T_R I] \\
\frac{\mathrm{d}[IS_N]}{\mathrm{d}t} &= \sigma[ES_N] + \sigma[FS_N] - (1-\psi)\delta[IS_N] - \psi\gamma[IS_N] - \tau([T_I IS_N] + [T_R IS_N]) - \beta(t)[IS_N I] \\
\frac{\mathrm{d}[IP]}{\mathrm{d}t} &= \sigma[EP] + \sigma[FP] - (1-\psi)\delta[IP] - \psi\gamma[IP] - \tau([T_I IP] + [T_R IP]) \\
&+ \tau([T_I SI] + [T_R SI]) - \beta(t)[IPI] - \upsilon[IP] \\
\frac{\mathrm{d}[IF]}{\mathrm{d}t} &= \sigma[EF] + \sigma[FF] - (1-\psi)\delta[IF] - \psi\gamma[IF] - \tau([T_I IF] + [T_R IF]) \\
&+ \beta(t)[IS_N I] + \beta(t)[IPI] - \tau([T_I FI] + [T_R FI]) - \sigma[IF]
\end{aligned}
$$

$$
\begin{aligned}
\frac{\mathrm{d}[RR]}{\mathrm{d}t} &= 2(1-\psi)\delta[IR] + 2\chi\tau([T_I ER] + [T_R ER]) + 2\omega[T_R R] + 2\upsilon[PR] \\
\frac{\mathrm{d}[RT_E]}{\mathrm{d}t} &= (1-\psi)\delta[IT_E] + \chi\tau([T_I ET_E] + [T_R ET_E]) + \omega[T_R T_E] + \upsilon[PT_E] \\
&+ (1-\chi)\tau([T_I ER] + [T_R ER]) + \tau([T_I FR] + [T_R FR]) - \sigma[RT_E] \\
\frac{\mathrm{d}[RT_I]}{\mathrm{d}t} &= (1-\psi)\delta[IT_I] + \chi\tau([T_I ET_I] + [T_R ET_I]) + \omega[T_R T_I] + \upsilon[PT_I] \\
&+ \psi\gamma[IR] + \tau([T_I IR] + [T_R IR]) - \delta[T_I R] + \sigma[T_E R] \\
\frac{\mathrm{d}[RT_R]}{\mathrm{d}t} &= (1-\psi)\delta[IT_R] + \chi\tau([T_I ET_R] + [T_R ET_R]) + \omega[T_R T_R] + \upsilon[PT_R] \\
&+ \delta[T_I R] - \omega[T_R R] \\
\frac{\mathrm{d}[RS_N]}{\mathrm{d}t} &= (1-\psi)\delta[IS_N] + \chi\tau([T_I ES_N] + [T_R ES_N]) + \omega[T_R S_N] + \upsilon[PS_N] - \beta(t)[IS_N R] \\
\frac{\mathrm{d}[RP]}{\mathrm{d}t} &= (1-\psi)\delta[IP] + \chi\tau([T_I EP] + [T_R EP]) + \omega[T_R P] + \upsilon[PP] \\
&+ \tau([T_I SR] + [T_R SR]) - \beta(t)[IPR] - \upsilon[RP] \\
\frac{\mathrm{d}[RF]}{\mathrm{d}t} &= (1-\psi)\delta[IF] + \upsilon[PF] \\
&+ \chi\tau([T_I EF] + [T_R EF]) + \omega[T_R F] + \beta(t)[IS_N R] + \beta(t)[IPR] - \sigma[RF] \\
&- \tau([T_I FR] + [T_R FR])
\end{aligned}
$$

$$
\begin{aligned}
\frac{\mathrm{d}[T_ET_E]}{\mathrm{d}t} &= 2(1-\chi)\tau([T_IET_E]+[T_RET_E])+2\tau([T_IFT_E]+[T_RFT_E])-2\sigma[T_ET_E]\\
\frac{\mathrm{d}[T_ET_I]}{\mathrm{d}t} &= (1-\chi)\tau([T_IET_I]+[T_RET_I])+\tau([T_IFT_I]+[T_RFT_I])-\sigma[T_ET_I]\\
&+ \tau([T_IIT_E]+[T_RIT_E])+\psi\gamma[IT_E]-\delta[T_ET_I]+\sigma[T_ET_E]\\
\frac{\mathrm{d}[T_ET_R]}{\mathrm{d}t} &= (1-\chi)\tau([T_IET_R]+[T_RET_R])+\tau([T_IFT_R]+[T_RFT_R])-\sigma[T_ET_R]\\
&+ \delta[T_IT_E]-\omega[T_ET_R]\\
\frac{\mathrm{d}[T_ES_N]}{\mathrm{d}t} &= (1-\chi)\tau([T_IES_N]+[T_RES_N])+\tau([T_IFS_N]+[T_RFS_N])\\
&- \sigma[T_ES_N]-\beta(t)[IS_NT_E]\\
\frac{\mathrm{d}[T_EP]}{\mathrm{d}t} &= (1-\chi)\tau([T_IEP]+[T_REP])+\tau([T_IFP]+[T_RFP])\\
&- \sigma[T_ES_N]+\tau([T_IST_E]+[T_RST_E])-\beta(t)[IPT_E]-\upsilon[PT_E]\\
\frac{\mathrm{d}[T_EF]}{\mathrm{d}t} &= (1-\chi)\tau([T_IEF]+[T_REF])+\tau([T_IFF]+[T_RFF])\\
&- 2\sigma[T_EF]+\beta(t)[IS_NT_E]+\beta(t)[IPT_E]\\
&- \tau([T_IFT_E]+[T_RFT_E])\\
\frac{\mathrm{d}[T_IT_I]}{\mathrm{d}t} &= 2\tau([T_IIT_I]+[T_RIT_I])-2\delta[T_IT_I]+2+\psi\gamma[IT_I]\\
&+ 2\sigma[T_ET_I]\\
\frac{\mathrm{d}[T_IT_R]}{\mathrm{d}t} &= \tau([T_IIT_R]+[T_RIT_R])-\delta[T_IT_R]+\psi\gamma[IT_R]\\
&+ \sigma[T_ET_R]\\
&+ \delta[T_IT_I]-\omega[T_RT_I]\\
\frac{\mathrm{d}[T_IS_N]}{\mathrm{d}t} &= \tau([T_IIS_N]+[T_RIS_N])-\delta[T_IS_N]+\psi\gamma[IS_N]-\beta(t)[IS_NT_I]\\
\frac{\mathrm{d}[T_IP]}{\mathrm{d}t} &= \tau([T_IIP]+[T_RIP])-\delta[T_IP]+\psi\gamma[IP]\\
&+ \tau([T_IST_I]+[T_RST_I])-\beta(t)[IPT_I]-\upsilon[PT_I]\\
\frac{\mathrm{d}[T_IF]}{\mathrm{d}t} &= \tau([T_IIF]+[T_RIF])-\delta[T_IF]+\psi\gamma[IF]+\beta(t)[IS_NT_I]\\
&- \sigma[T_IF]-\tau([T_IFT_I]+[T_RFT_I])+\beta(t)[IPT_I]\\
\frac{\mathrm{d}[T_RT_R]}{\mathrm{d}t} &= 2\delta[T_IT_R]-2\omega[T_RT_R]\\
\frac{\mathrm{d}[T_RS_N]}{\mathrm{d}t} &= \delta[T_IS_N]-\omega[T_RS_N]-\beta(t)[IS_NT_R]\\
\frac{\mathrm{d}[T_RP]}{\mathrm{d}t} &= \delta[T_IP]-\omega[T_RP]+\tau([T_IST_R]+[T_RST_R])-\beta(t)[IPT_R]-\upsilon[PT_R]\\
\frac{\mathrm{d}[T_RF]}{\mathrm{d}t} &= \delta[T_IF]-\omega[T_RF]+\beta(t)[IS_NT_R]\\
&- \sigma[T_RF]-\tau([T_IFT_R]+[T_RFT_R])+\beta(t)[IPT_R]\\
\frac{\mathrm{d}[S_NS_N]}{\mathrm{d}t} &= -2\beta(t)[IS_NS_N]\\
\frac{\mathrm{d}[S_NP]}{\mathrm{d}t} &= -\beta(t)[IS_NP]+\tau([T_ISS_N]+[T_RSS_N])-\beta(t)[IPS_N]-\upsilon[PS_N]
\end{aligned}
$$

$$
\begin{aligned}
\frac{\mathrm{d}[S_NF]}{\mathrm{d}t} &= -\beta(t)[IS_NF] + \beta(t)[IS_NS_N] - \sigma[S_NF] - \tau([T_IFS_N] + [T_RFS_N]) + \beta(t)[IPS_N] \\
\frac{\mathrm{d}[PP]}{\mathrm{d}t} &= 2\tau([T_ISP] + [T_RSP]) - 2\beta(t)[IPP] - 2\upsilon[PP] \\
\frac{\mathrm{d}[PF]}{\mathrm{d}t} &= \tau([T_ISF] + [T_RSF]) - \beta(t)[IPF] - \upsilon[PF] \\
&+ \beta(t)[IS_NP] - \sigma[FF] - \tau([T_IFF] + [T_RFF]) + \beta(t)[IPP] \\
\frac{\mathrm{d}[FF]}{\mathrm{d}t} &= 2\beta(t)[IS_NF] - 2\sigma[FF] - 2\tau([T_IFF] + [T_RFF]) + 2\beta(t)[IPF]
\end{aligned}
$$

References

1. Legrand J, Grais RF, Boelle PY, Valleron AJ, Flahault A (2007) Understanding the dynamics of Ebola epidemics. Epidemiology and infection 135: 610–21.

2. Chowell G, Hengartner N, Castillo-Chavez C, Fenimore P, Hyman J (2004) The basic reproductive number of ebola and the effects of public health measures: the cases of congo and uganda. Journal of Theoretical Biology 229: 119 - 126.

3. Smieszek T, Fiebig L, Scholz RW (2009) Models of epidemics: when contact repetition and clustering should be included. Theoretical biology & medical modelling 6: 11.

4. Lekone PE, Finkenstädt BF (2006) Statistical inference in a stochastic epidemic SEIR model with control intervention: Ebola as a case study. Biometrics 62: 1170–7.

5. Rand D (1999) Correlation equations and pair approximations for spatial ecologies. Advanced ecological theory: principles and applications 100.

6. World Health Organization (2014). Are the Ebola outbreaks in Nigeria and Senegal over? URL `http://www.who.int/mediacentre/news/ebola/14-october-2014/en/`.

7. Centers for Disease Control (2004). Contact Tracing: Contact tracing can stop Ebola in its tracks. URL `http://www.cdc.gov/vhf/ebola/outbreaks/what-is-contact-tracing.html`.

8. World Health Organization (2014). Ebola data and statistics: Liberia. URL `http://apps.who.int/gho/data/node.ebola-sitrep.ebola-country-LBR?lang=en`.

9. World Health Organization (2014). Ebola data and statistics: Sierra Leone. URL `http://apps.who.int/gho/data/node.ebola-sitrep.ebola-country-SLE?lang=en`.

10. Meltzer M, Atkins C, Santibanez S, Knust B, Peterson B, et al. (2014) Estimating the Future Number of Cases in the Ebola Epidemic Liberia and Sierra Leone , 2014 2015. Technical Report 3, Centers for Disease Control and Prevention. URL `http://www.cdc.gov/mmwr/preview/mmwrhtml/su6303a1.htm`.

11. Dhillon RS, Srikrishna D, Sachs J (2014) Controlling Ebola: next steps. Lancet 384: 1409–1411.

12. Emma Farge UF (2014). Ebola end game in sight as new cases fall sharply in Sierra Leone. URL `http://www.reuters.com/article/2015/01/21/health-ebola-leone-idUSL6N0UT2Z120150121`.

13. Government of Canada (2014). Operation SIRONA Task Force commences operations in fighting Ebola outbreak - Canadian Armed Forces personnel working with British forces in Sierra Leone. URL `http://news.gc.ca/web/article-en.do?nid=917269`.

14. National Defence and the Canadian Armed Forces (2014). Operation SIRONA. URL `http://www.forces.gc.ca/en/operations-abroad/op-sirona.page`.

15. MathWorks (2004). Optimization toolbox: lsqnonlin. URL `http://www.mathworks.com/help/optim/ug/lsqnonlin.html`.

16. Gire SK, Goba A, Andersen KG, Sealfon RSG, Park DJ, et al. (2014) Genomic surveillance elucidates Ebola virus origin and transmission during the 2014 outbreak. Science 1369.
