# Supplementary material for: Harnessing Case Isolation and Ring Vaccination to Control Ebola
Source: PLoS Negl Trop Dis. 2015 May 29;9(5):e0003794. doi: 10.1371/journal.pntd.0003794 (PMC4449200; doi:10.1371/journal.pntd.0003794)
Supplement: S2 Text — (PDF) [file pntd.0003794.s002.pdf]

Text S2: Extension of sensitivity analysis regarding mixing patterns and vaccine characteristics

Chad Wells, Dan Yamin, Martial L. Ndeffo-Mbah, Natasha Wenzel, Stephen G. Gaffney, Jeffrey P. Townsend, Lauren Ancel Meyers, Mosoka Fallah, Tolbert G. Nyenswah, Frederick L. Altice, Katherine E. Atkins, and Alison P. Galvani

Contact Tracing Efficacy

Our sensitivity analysis shows that with contact tracing efficacies ranging from 5% to 50%, case isolation alone is predicted to result in 3,874 to 3,988 confirmed cases in Liberia and 10,246 to 11,048 in Sierra Leone. If ring vaccination was implemented with a prophylactic vaccine in addition to case isolation, the number of confirmed cases is predicted to range from 3,871 to 3,987 in Liberia and from 10,228 to 11,039 in Sierra Leone, as vaccine efficacy varies between 30% and 100% (Fig. 2). If the vaccine confers post-exposure protection, the number of confirmed cases could range from 3,747 to 3,982 in Liberia and from 9,686 to 11,029 in Sierra Leone, as vaccine efficacy varies between 30% and 100% (Fig. 2).

A vaccine that confers both prophylactic and post-exposure protection could substantially reduce the number of isolations compared to a prophylactic vaccine (S4 Fig.). Specifically, a vaccine that confers post-exposure protection could reduce the number of symptomatic individuals identified by contact tracing who require hospitalization by up to 50 cases in Liberia and by 165 cases in Sierra Leone (S4 Fig.).

Average Degree

The marginal benefit of ring vaccination was found to be lower in populations with fewer contacts per individual. Specifically, the greatest marginal benefit of ring vaccination with a prophylactic vaccine in a population with fewer contacts per individual ($k = 5.74$) was 0.28% in Liberia and 0.63% in Sierra Leone, compared to the 0.52% in Liberia and 1.27% in Sierra Leone for a population with more contacts per individual ($k = 10$) (S2 Fig.).

Similarly, the marginal benefit of increasing the contact tracing efficacy for both case isolation and the combined intervention is higher for greater numbers of contacts per individual. For example, increasing the contact tracing efficacy for the combined intervention from 5% to 40% in Liberia is predicted to reduce the Ebola incidence by as much as 3.8% in a population with fewer contacts per individual ($k = 5.74$) and as much as 5.6% when the number of contacts per individual is greater ($k = 10$) (S6 Fig.). In Sierra Leone, increasing the contact tracing efficacy for the combined intervention from 5% to 40% reduced the incidence by as much as 7.9% in a population with fewer contacts per individual and as much as 12.3% in a population with a greater number of contacts per individual (S6 Fig.).

Clustering

The marginal benefit of ring vaccination with a prophylactic vaccine was greater in highly clustered populations compared to moderately and loosely clustered populations. Specifically, the maximum marginal benefit of adding ring vaccination in Liberia was 0.23% in a loosely clustered population ($\phi = 0.10$), 0.28% in a moderately clustered population ($\phi = 0.21$) and 0.32% in a highly clustered population ($\phi = 0.40$). In Sierra Leone, the maximum marginal benefit of adding ring vaccination was predicted to be 0.54% in a loosely clustered population, 0.63% in a moderately clustered population and 0.61% in a highly cluster population (S5 Fig.).

Clustering was shown to reduce the marginal benefit of increasing the contact tracing efficacy for both case isolation and a combined intervention (S7 Fig.). For example, increasing the contact tracing efficacy for a combined intervention from 5% to 40% in Liberia reduces Ebola incidence by as much as 6.7% in a loosely clustered population ($\phi = 0.10$), by 3.8% in a moderately clustered population ($\phi = 0.21$), and by 1.9% in a highly clustered population ($\phi = 0.40$) (S7 Fig.). In Sierra Leone, increasing the contact tracing efficacy for a combined intervention from 5% to 40% reduces Ebola incidence by as much as 14.2% in a loosely clustered population, by 7.9% in a moderately clustered population, and by 3.2% in a highly clustered population (S7 Fig.).
