# Supplementary figures and images for: Harnessing Case Isolation and Ring Vaccination to Control Ebola
Source: PLoS Negl Trop Dis. 2015 May 29;9(5):e0003794. doi: 10.1371/journal.pntd.0003794 (PMC4449200; doi:10.1371/journal.pntd.0003794)

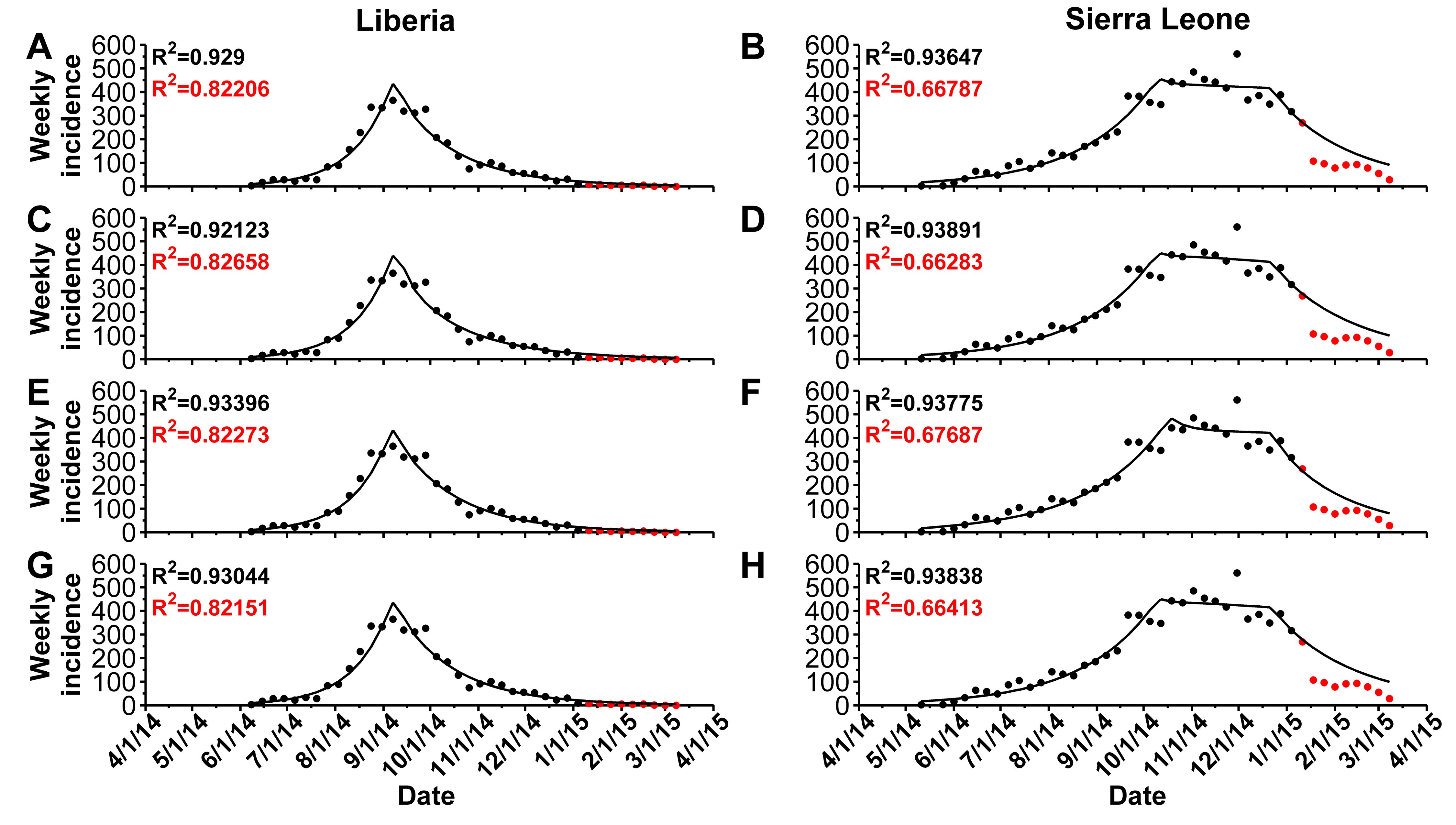

Supplement: S1 Fig — The model was fit to confirmed incidence data (black dots) from June 8, 2014 to January 4, 2015 for Liberia and May 11, 2014 to January 4, 2015 for Sierra Leone. We forecasted confirmed incidence until March 8, 2015 (red points). For each fit, we calculated the correlation fit value (R 2) for the fitted portion (black) and the forecasted portion (red) of the data. (TIFF) [file pntd.0003794.s003.tiff]

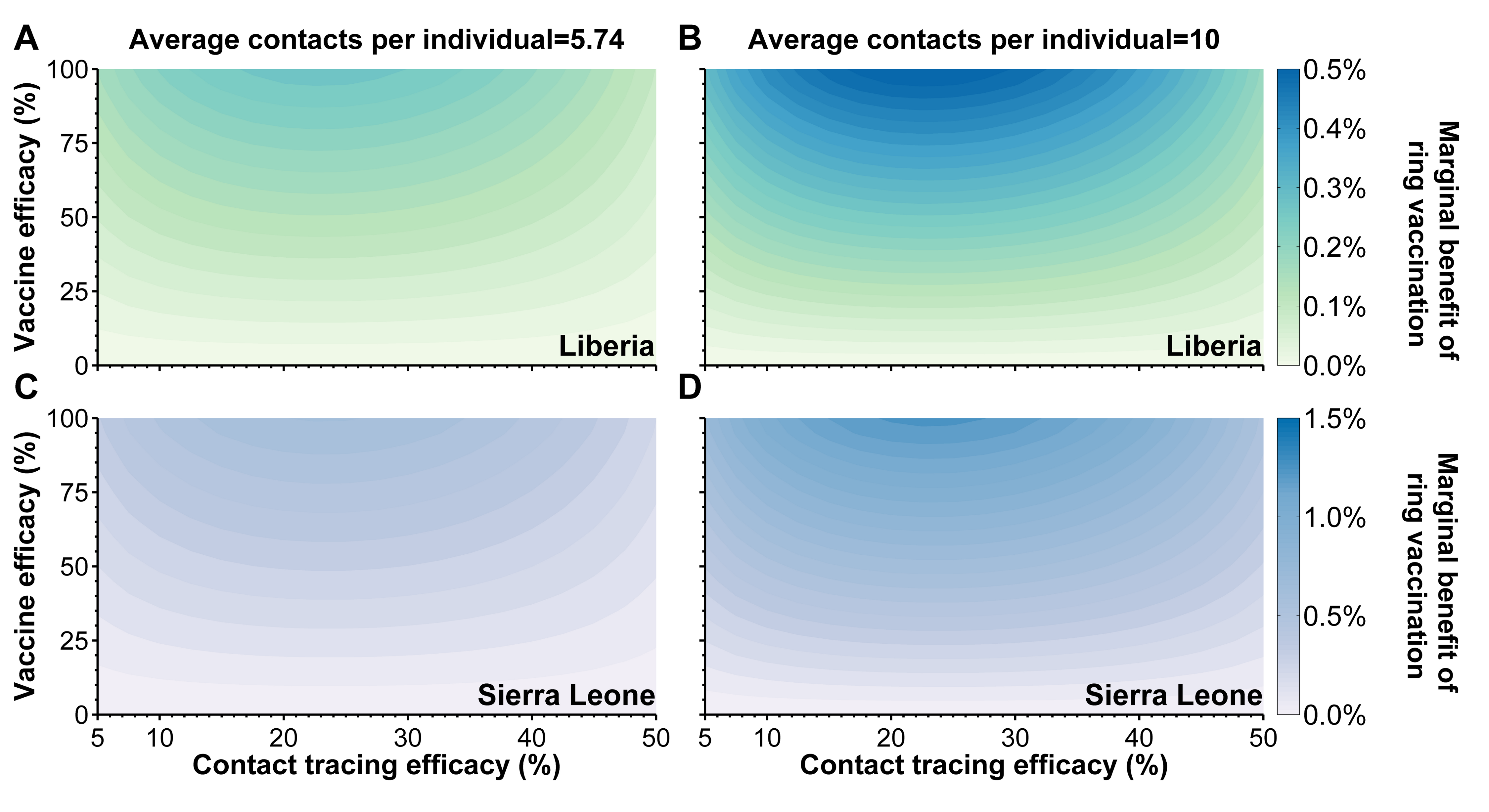

Supplement: S2 Fig — The model was fit using A), C) k = 5.74 and B), D) k = 10, with a clustering coefficient of ϕ = 0.21. A vaccine efficacy of zero would correspond to the implementation of case isolation only. The marginal benefit was calculated from the initiation of intervention scale up to the end of the epidemic. (TIFF) [file pntd.0003794.s004.tiff]

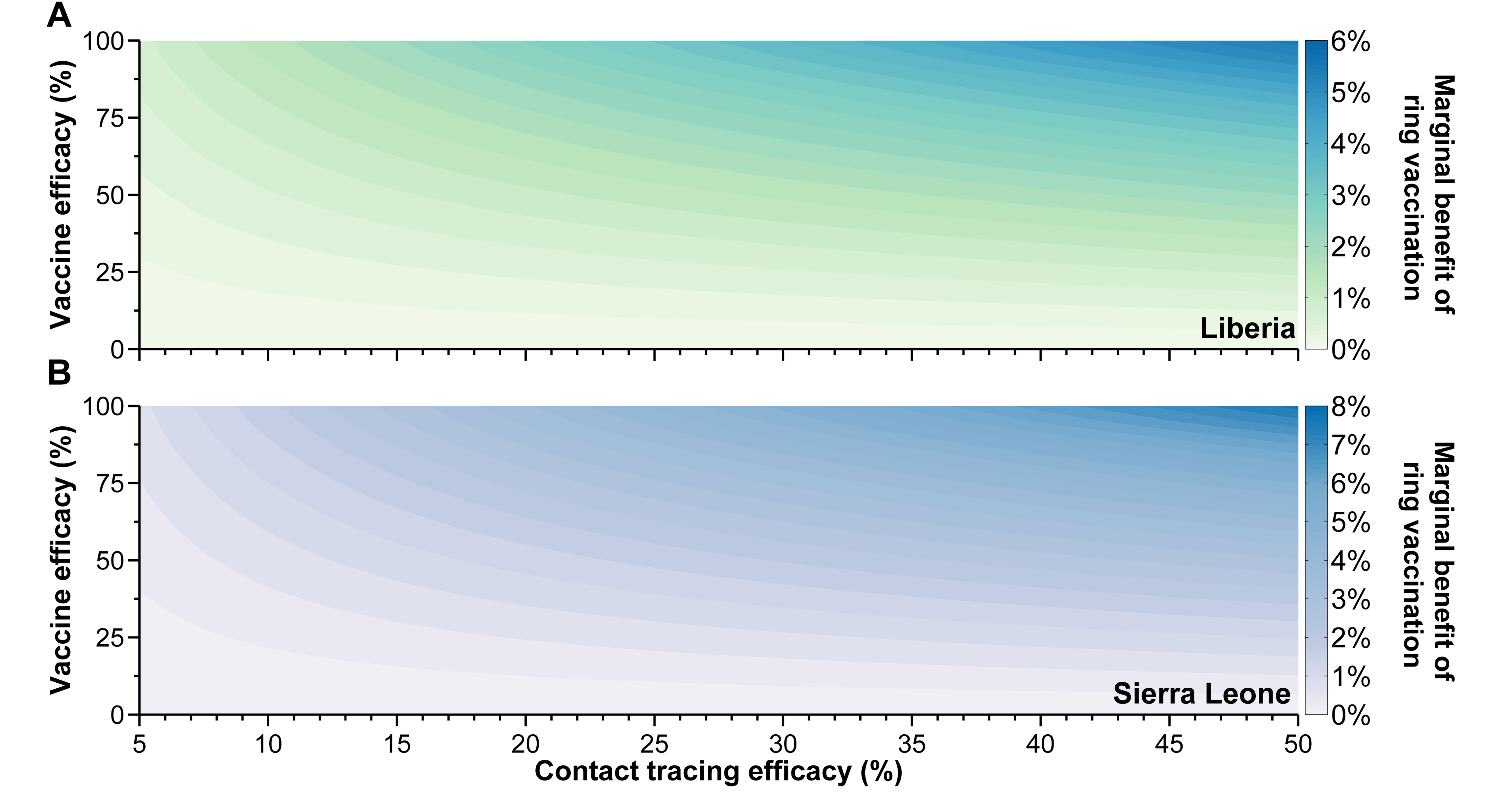

Supplement: S3 Fig — The model was fit using k = 5.74 with a clustering coefficient of ϕ = 0.21. A vaccine efficacy of zero would correspond to the implementation of case isolation only. The marginal benefit was calculated from the initiation of intervention scale up to the end of the epidemic. (TIFF) [file pntd.0003794.s005.tiff]

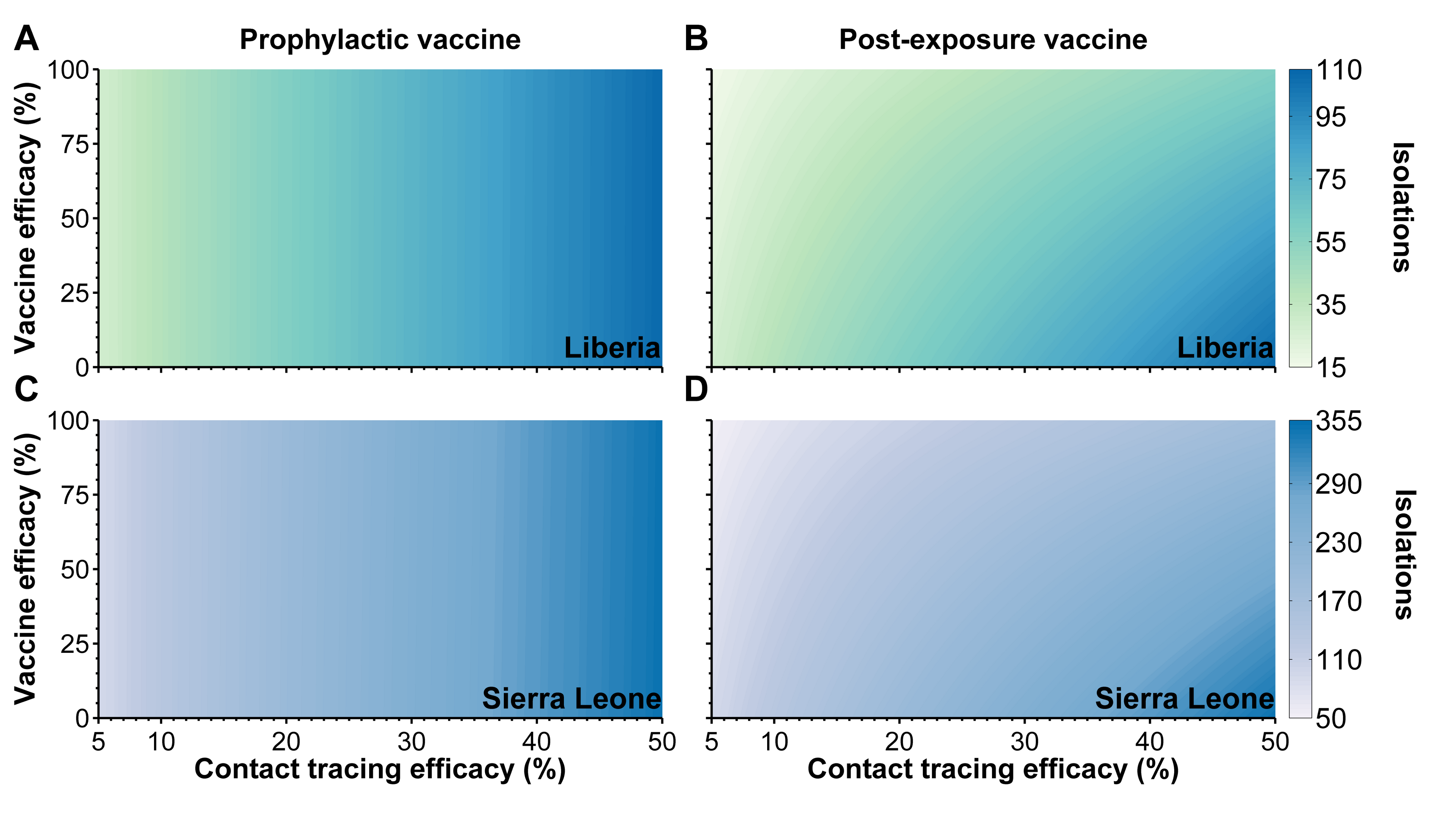

Supplement: S4 Fig — A vaccine efficacy of zero would correspond to the implementation of case isolation only. The model was fit using k = 5.74 and with a clustering coefficient of ϕ = 0.21. (TIFF) [file pntd.0003794.s006.tiff]

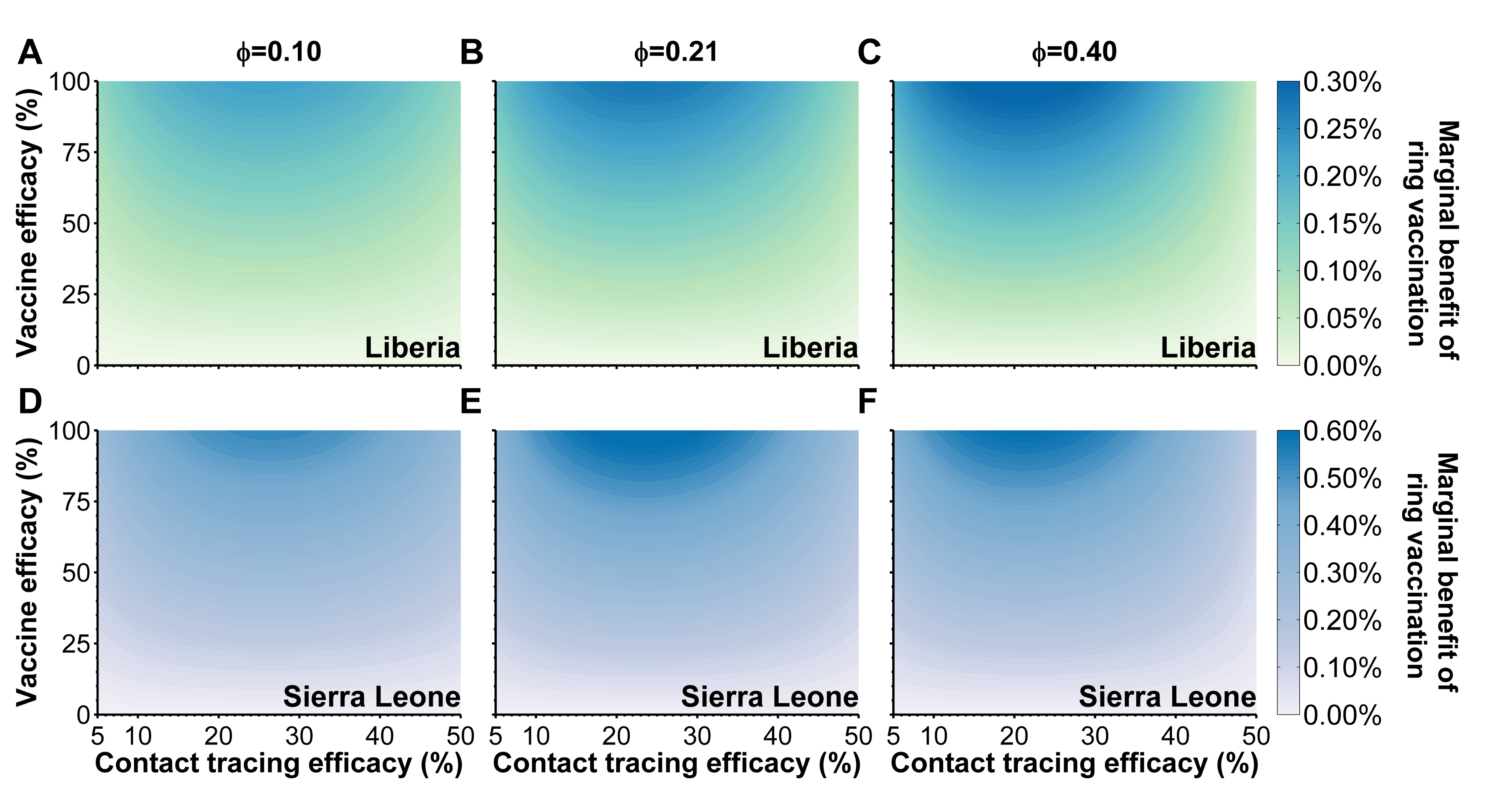

Supplement: S5 Fig — The model was fit using k = 5.74 with a clustering coefficient of A), D) ϕ = 0.10, B), E) ϕ = 0.21, and C), F) ϕ = 0.40. A vaccine efficacy of zero would correspond to the implementation of case isolation only. The marginal benefit was calculated from the initiation of intervention scale up to the end of the epidemic. (TIFF) [file pntd.0003794.s007.tiff]

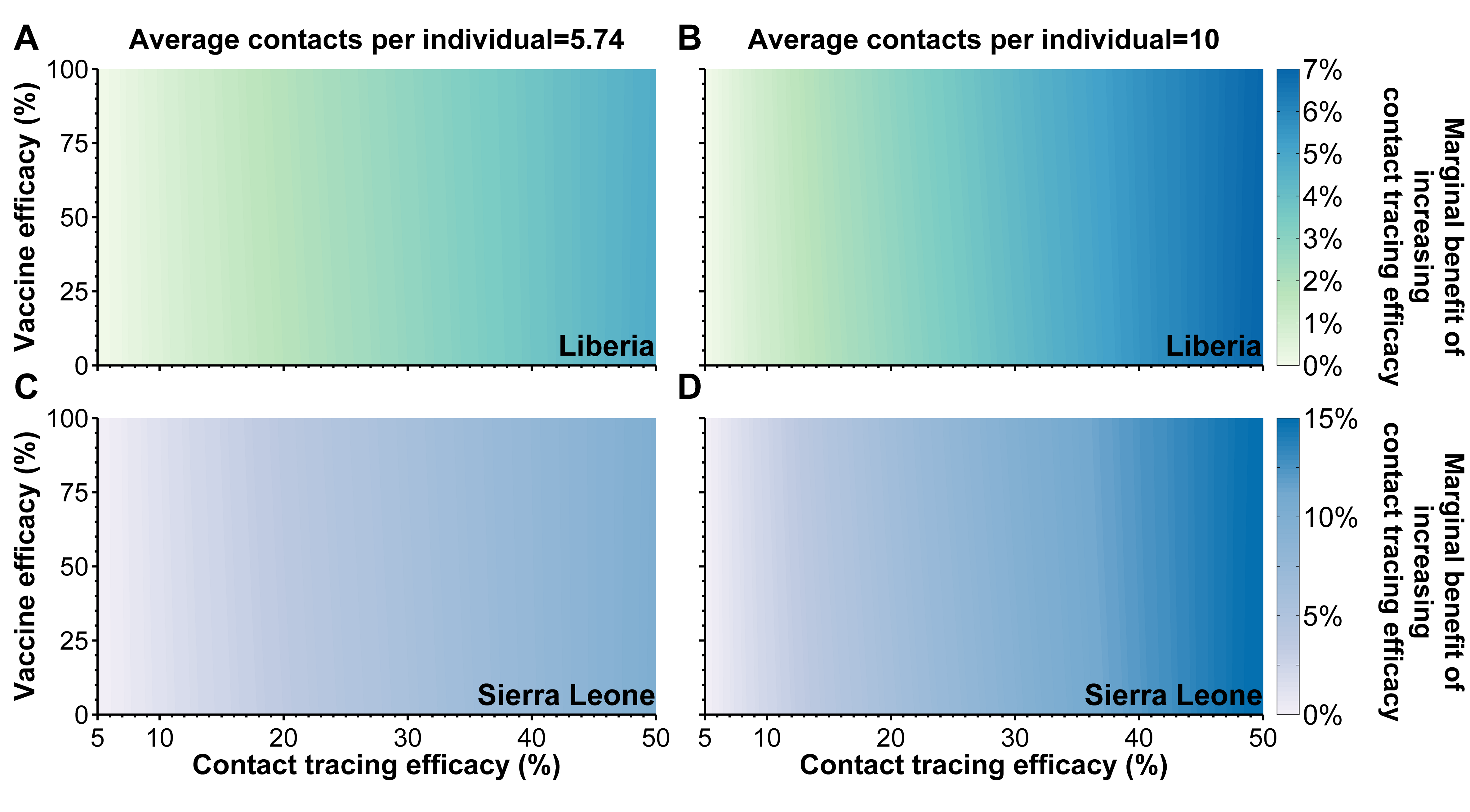

Supplement: S6 Fig — The model was fit using A), C) k = 5.74 and B), D) k = 10, with a clustering coefficient of ϕ = 0.21. A vaccine efficacy of zero would correspond to the implementation of case isolation only. The marginal benefit was calculated from the initiation of intervention scale up to the end of the epidemic. (TIFF) [file pntd.0003794.s008.tiff]

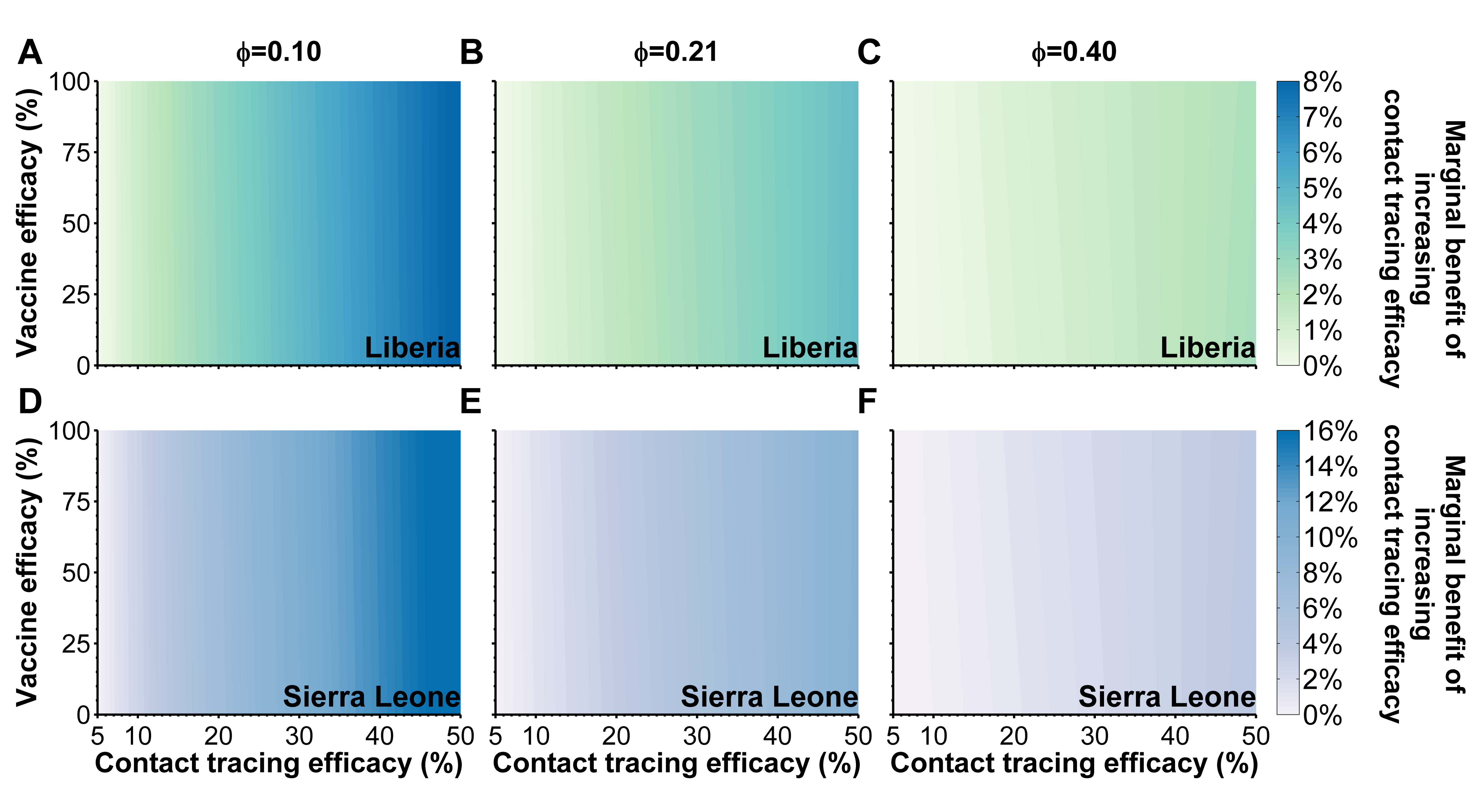

Supplement: S7 Fig — The model was fit using k = 5.74 with a clustering coefficient of A), D) ϕ = 0.10, B), E) ϕ = 0.21, and C), F) ϕ = 0.40. A vaccine efficacy of zero would correspond to the implementation of case isolation only. The marginal benefit was calculated from the initiation of intervention scale up to the end of the epidemic. (TIFF) [file pntd.0003794.s009.tiff]

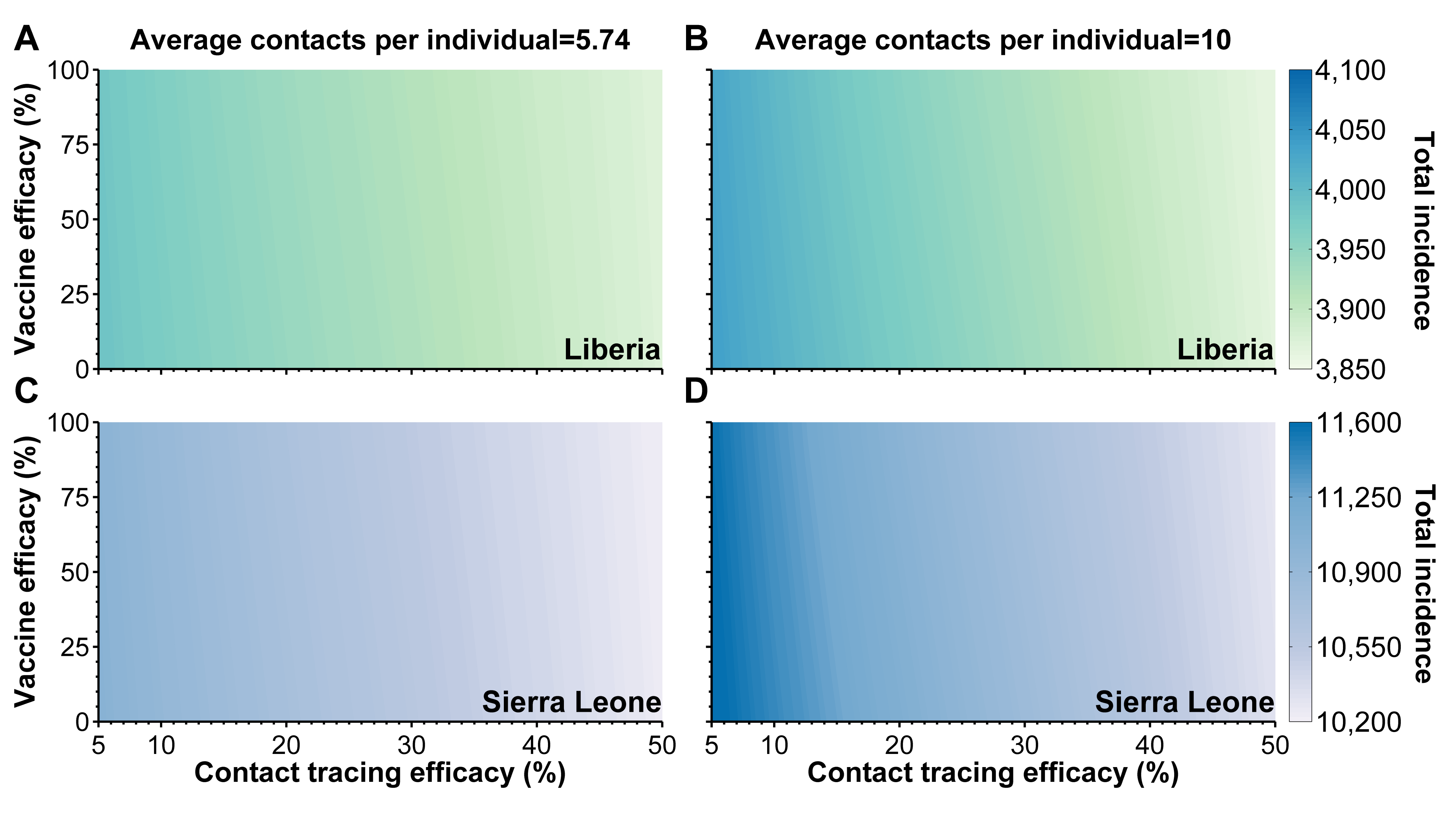

Supplement: S8 Fig — The model was fit using A), C) k = 5.74 and B), D) k = 10, with a clustering coefficient of ϕ = 0.21. A vaccine efficacy of zero would correspond to the implementation of case isolation only. (TIFF) [file pntd.0003794.s010.tiff]

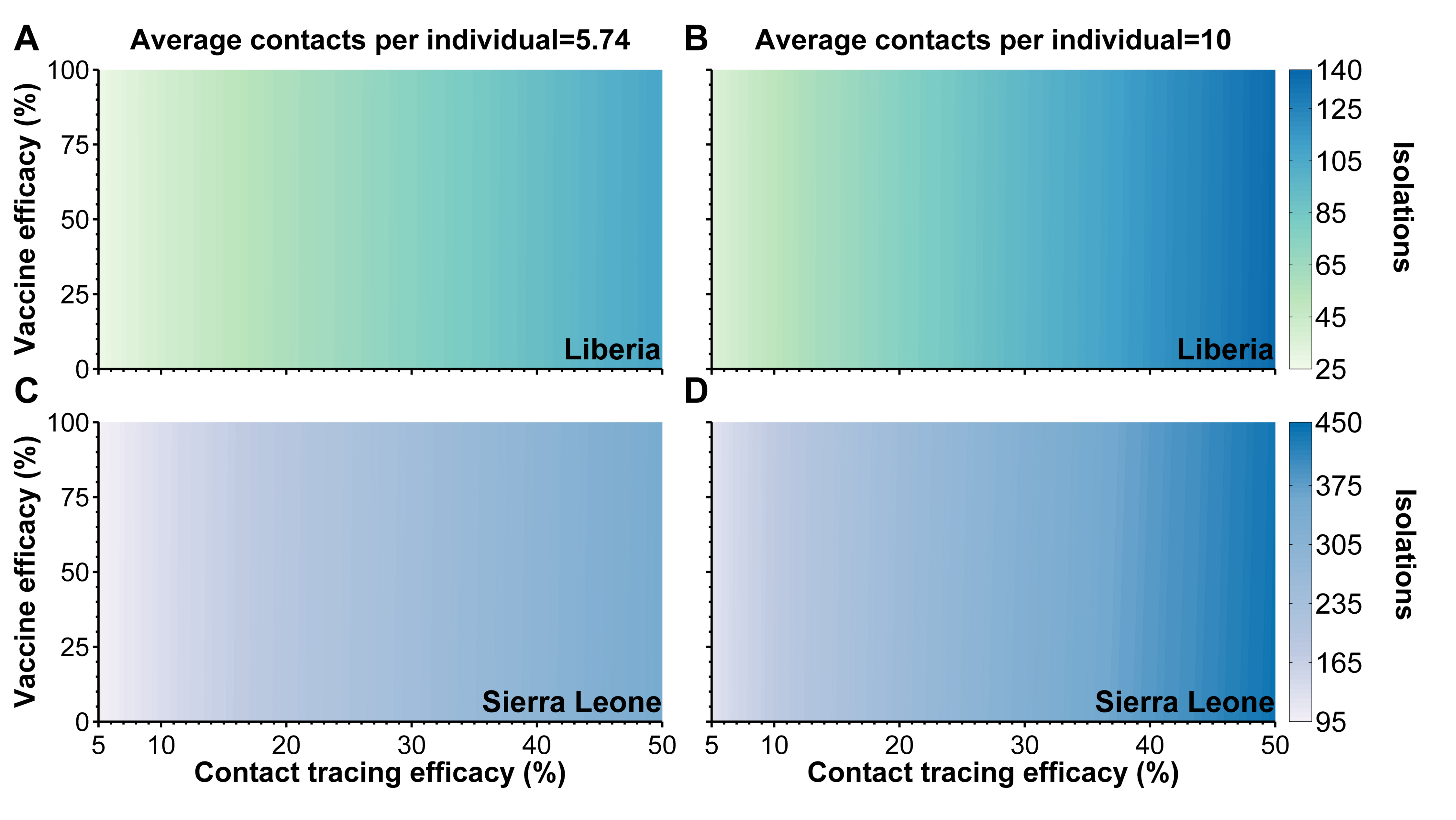

Supplement: S9 Fig — The model was fit using A), C) k = 5.74 and B), D) k = 10, with a clustering coefficient of ϕ = 0.21. A vaccine efficacy of zero would correspond to the implementation of case isolation only. (TIFF) [file pntd.0003794.s011.tiff]

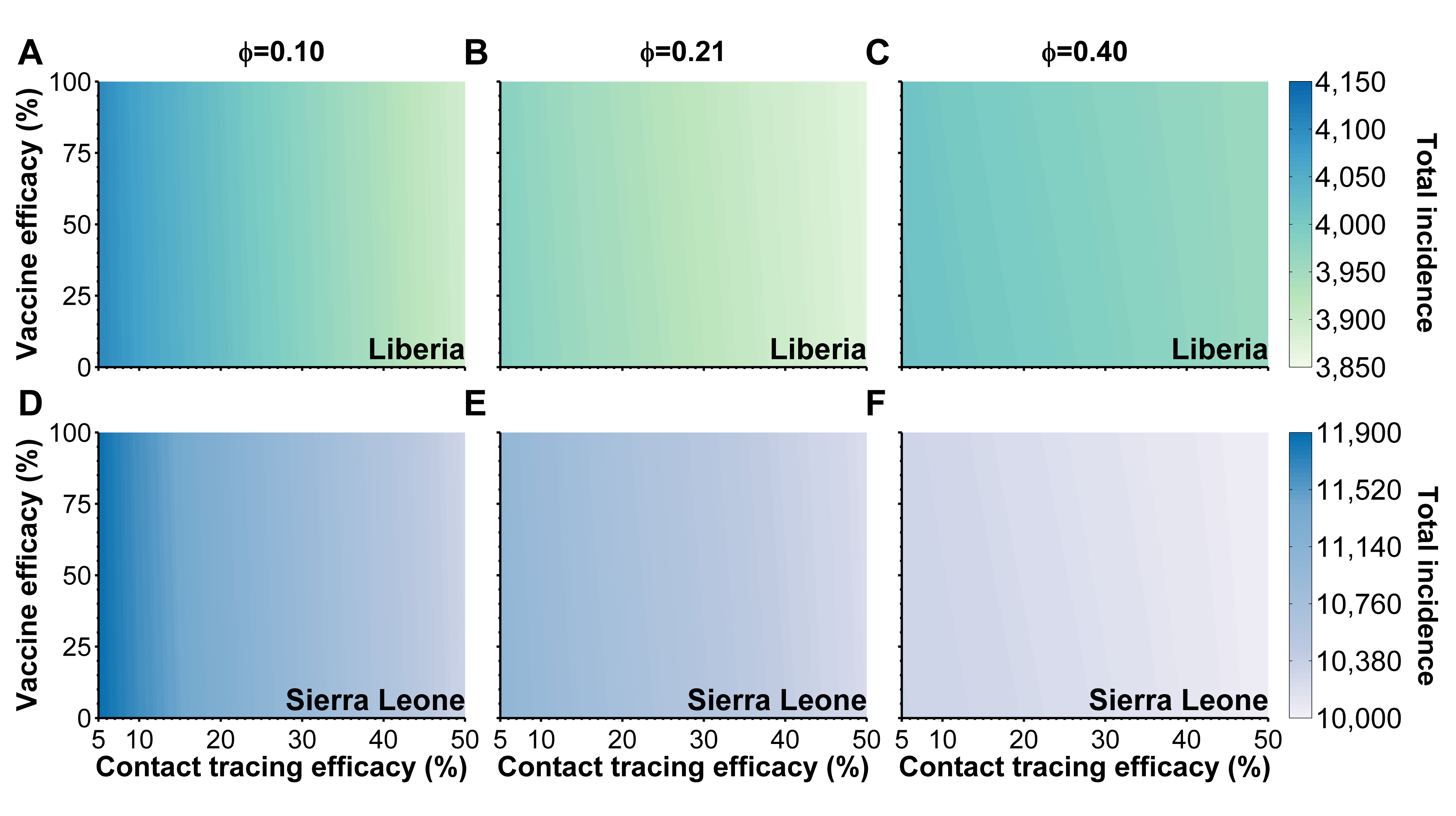

Supplement: S10 Fig — The model was fit using k = 5.74 with a clustering coefficient of A), D) ϕ = 0.10, B), E) ϕ = 0.21, and C), F) ϕ = 0.40. A vaccine efficacy of zero would correspond to the implementation of case isolation only. (TIFF) [file pntd.0003794.s012.tiff]

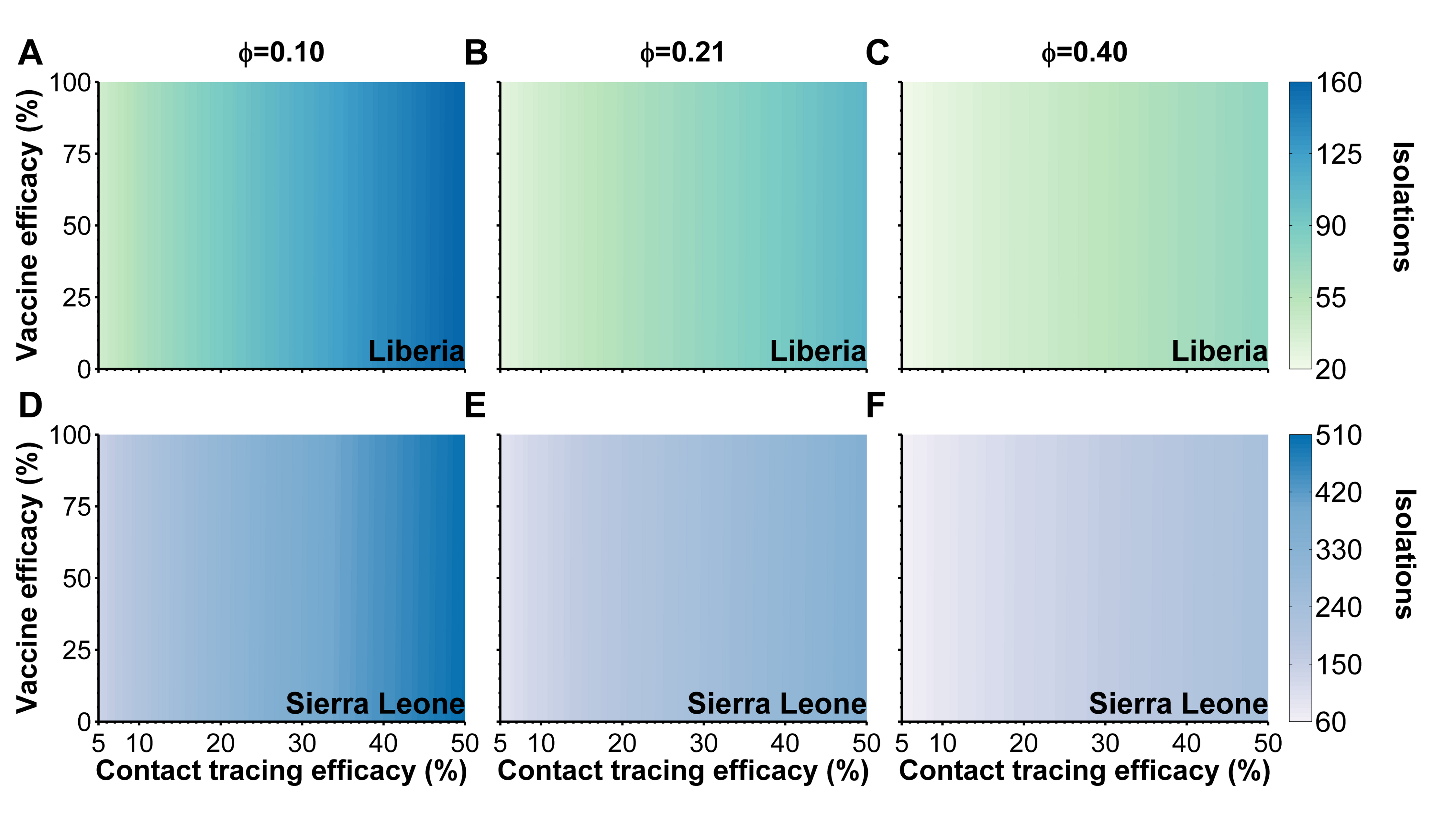

Supplement: S11 Fig — The model was fit using k = 5.74 with a clustering coefficient of A), D) ϕ = 0.10, B), E) ϕ = 0.21, and C), F) ϕ = 0.40. A vaccine efficacy of zero would correspond to the implementation of case isolation only. (TIFF) [file pntd.0003794.s013.tiff]

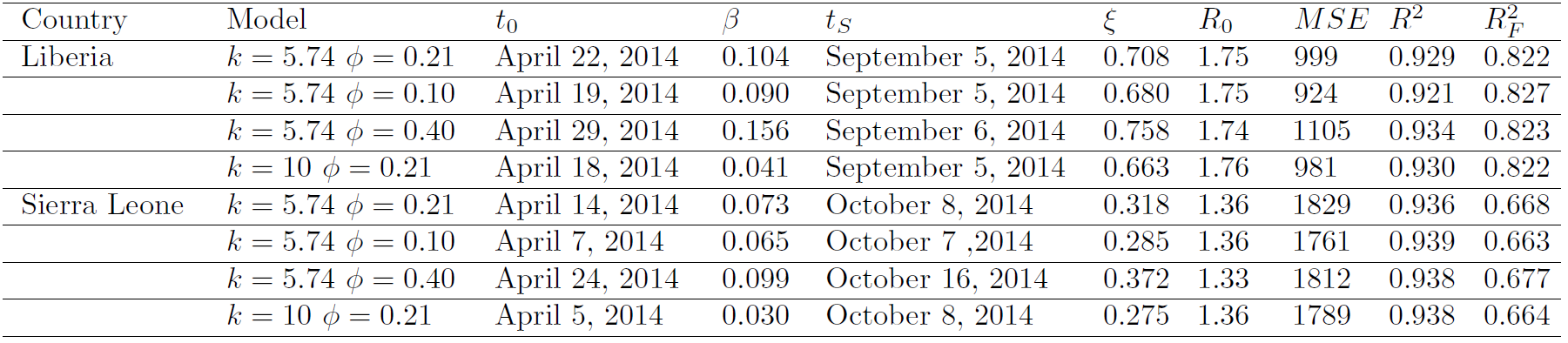

Supplement: S1 Table — Using confirmed incidence data for Liberia and Sierra Leone, we estimated the rate of transmission per infectious contact (β), the date of Ebola emergence into the population (t 0), the initiation of intervention scale up (t S), and reduced transmission mediated by factors other than intervention (ξ) for a given average number of contacts (k) and clustering coefficient (ϕ). For each scenario we provide the basic reproductive number (R 0), the mean square error (MSE), and the correlation fit value for both the fitted portion (R 2) and the forecasted portion (RF2) of the epidemic trajectory. (TIFF) [file pntd.0003794.s014.tiff]
